# Probabilistic quotient’s work and pharmacokinetics’ contribution: countering size effect in metabolic time series measurements

**DOI:** 10.1186/s12859-022-04918-1

**Published:** 2022-09-16

**Authors:** Mathias Gotsmy, Julia Brunmair, Christoph Büschl, Christopher Gerner, Jürgen Zanghellini

**Affiliations:** 1 Department of Analytical Chemistry, Faculty of Chemistry, University of Vienna, Vienna, Austria; 2 Vienna Doctoral School in Chemistry, University of Vienna, Vienna, Austria; 3 Joint Metabolome Facility, University and Medical University of Vienna, Vienna, Austria

**Keywords:** Metabolomics, Finger Sweat, Blood Plasma, PKM, PQN

## Abstract

Metabolomic time course analyses of biofluids are highly relevant for clinical diagnostics. However, many sampling methods suffer from unknown sample sizes, commonly known as size effects. This prevents absolute quantification of biomarkers. Recently, several mathematical post acquisition normalization methods have been developed to overcome these problems either by exploiting already known pharmacokinetic information or by statistical means. Here we present an improved normalization method, MIX, that combines the advantages of both approaches. It couples two normalization terms, one based on a pharmacokinetic model (PKM) and the other representing a popular statistical approach, probabilistic quotient normalization (PQN), in a single model. To test the performance of MIX, we generated synthetic data closely resembling real finger sweat metabolome measurements. We show that MIX normalization successfully tackles key weaknesses of the individual strategies: it (i) reduces the risk of overfitting with PKM, and (ii), contrary to PQN, it allows to compute sample volumes. Finally, we validate MIX by using real finger sweat as well as blood plasma metabolome data and demonstrate that MIX allows to better and more robustly correct for size effects. In conclusion, the MIX method improves the reliability and robustness of quantitative biomarker detection in finger sweat and other biofluids, paving the way for biomarker discovery and hypothesis generation from metabolomic time course data.

**Supplementary Information:**

The online version contains supplementary material available at 10.1186/s12859-022-04918-1.

## Introduction

In recent years, the analysis of the sweat metabolome has received increased attention from several fields of study [1–3]. For example, sweat has been in the focus of forensic scientists since it is possible to analyze metabolomic profiles of finger-prints that have been found (e.g., at a crime scene) [4]. Also, drug testing can easily be performed on sweat samples. One advantage of this method is to not only identify already illegal substances but their metabolic degradation products as well, thereby allowing scientist to distinguish between drug consumption and mere contact [1]. Another application of sweat metabolomics is in diagnostics for personalized medicine, where the focus is put on discerning metabolic states of the body and trying to optimize nutrition and treatment based upon information of biomarkers in sweat [5–7].

Sweat metabolomics offers several technical advantages. Firstly, sweat is a rich source of biomolecules and thus offers great potential for biomarker discovery [8, 9]. Secondly, sweat sampling is easy compared to sampling other biofluids (e.g., blood or urine). Moreover, it is non-invasive and can, in principle, be rapidly repeated.

Several sampling methods have been developed [2, 3, 9, 10]. However, most of them work in a very similar manner: a water absorbing material is put onto the skin’s surface to collect sweat for some (short) time. Sweat metabolites are subsequently extracted from this material and analyzed [3, 10]. Methods differ, however, in if and how they induce sweating. Some methods induce increased sweating by physical exercise [9] or chemical stimulation [2], whereas in other studies no sweat induction is performed and the natural sweat rate is sufficient for metabolomic analysis [3, 11].

Regardless of the exact sampling method, most of the above mentioned studies suffer one major drawback. The sweat flux is highly variable, depending not only on interindividual differences but also on body location, temperature, humidity, exercise, and further factors that may change multiple times over the course of one day [12, 13]. For example, even with conservative estimates a variability of sweat flux, $q_\text {sweat}$, on the finger-tips between 0.05 and 1 mg cm$^{-2}$ min$^{-1}$ needs to be accounted for [13–16]. This is a major challenge for comparative or quantitative studies, which has been acknowledged by many, e.g. [1, 4, 8, 17–19], however only actively approached by few – most notably [9].

The key problem is associated to the fact that often one is interested in the true metabolite concentrations, $\mathbf {C}\in {\mathbb R}^{n_{\text {metabolites}}}$, of ${n_{\text {metabolites}}}$ metabolites, which is obscured by an unknown and time-dependent sweat flux. Thus, the measured metabolites’ intensities are not proportional to $\mathbf {C}$ but to the metabolite mass vector, $\widetilde{\mathbf {M}}\in {\mathbb R}^{n_{\text {metabolites}}}$,

1 $$\begin{aligned} \widetilde{\mathbf {M}}(t) = a_\text {sample}\int _{t-\tau }^{t} \mathbf {C}(t')\ q_\text {sweat}(t')\ \text {d}t'. \end{aligned}$$

Here $a_\text {sample}$ and $\tau$ denote the surface area of skin that is sampled and the time it takes to collect one sample, respectively. We emphasize that throughout the manuscript, the mass of a metabolite is defined as the measured abundance of the metabolite in a measured sample and neither as the molar mass or mass to charge ratio. Moreover, we acknowledge that without a calibration curve, the measured abundances have an arbitrary peak-area unit and are thus strictly neither absolute masses nor concentrations. The proportionality constant that scales measured intensities to mass units is determined by the calibration curve. The proper calibration curve is not further discussed here but is assumed to be linear and available when applicable.

Metabolic concentration shifts happen in the span of double-digit minutes to hours, whereas sampling times are usually low single-digit minutes, therefore it is possible to assume that $\mathbf {C}$ changes little over the integration time $\tau$ [20]. Thus (1) simplifies to

2a $$\begin{aligned} \widetilde{\mathbf {M}}(t)&\approx \mathbf {C}(t) \ V(t), \end{aligned}$$

with an unknown sweat volume during sampling

2b $$\begin{aligned} V(t)&:= a_\text {sample}\int _{t-\tau }^{t} q_\text {sweat}(t')\ \text {d}t', \end{aligned}$$

and the problems reads: given $\widetilde{\mathbf {M}}$, how can we compute $\mathbf {C}$ if we don’t know $V$?

The need to calculate absolute metabolite concentrations from small biological samples of unknown volume is not unique to sweat metabolomics but known throughout untargeted metabolomics. The problem is commonly referred to as size effects [21]. For the sake of consistency with previous publications on this topic, we will use the term “size effects“ throughout this publication. We emphasize that in this context, it specifically refers to perceived differences in measured abundances due to changing sample volumes and/or dilutions and not to effects of different numbers of measurements per sample, also referred to as sample size effects [22].

Three strategies have been developed to tackle size effects:

*Direct sweat volume measurement.* Measuring $V$, for instance via microfluidics [9, 23, 24], is the most straight forward method to solve (2) and typically very accurate with minimally required volumes in the range of $\sim 5$ to 100 $\upmu$L [9, 23, 24]. However, in the case of sweat sampling, it may take quite some time, large sample areas, or increased (i.e., induced) sweating to collect enough sweat for robust volume quantification. Another alternative is the volume estimation via paired standards [25], however, such a method increases the complexity of sample preparation. Either option would impede fast and easy sample collection and analysis.

*Indirect sweat volume computation.* If the chemical kinetics of targeted metabolite concentrations are known, then kinetic parameters and the sweat volume at each time point can be simultaneously determined by fitting the measured mass vector to Eq. 2. Recently, we used this strategy to computationally resolve not only sample volumes in the nL to single digit $\upmu$L-range but also accurately quantify personalized metabolic response patterns upon caffeine ingestion [20]. Albeit feasible for the determination of individual differences with knowledge of reaction kinetics, this method quickly becomes unconstrained when too little prior information is available. Therefore, it is not suited for the discovery of unknown reaction kinetics. Moreover, this method requires several sampling time points to allow modeling the kinetics of different metabolites, thereby decreasing the simplicity of sampling.

*Statistical normalization.* With this approach the aim is to normalize the mass vector by the apparent mass of a marker that scales proportionally to the sample volume so that the ratio becomes (at least approximately) independent of the sample volume. Various strategies have been developed for untargeted metabolomics; for example, normalization by total measured signal [26], and singular value decomposition-based normalization [27]. However, one of the best performing methods – probabilistic quotient normalization (PQN) – simply assumes that the median of the ratio of two apparent mass vectors is proportional to the sample volume [21, 28–30]. Although PQN does not allow one to compute sample volumes *per se*, it enables one to assess differential changes [28].

In this study, we explore the performance of three different normalization methods on synthetic data. We illustrate the disadvantages of two previously published methods only focusing on either targeted or untargeted metabolites, respectively. A third normalization method is developed by combining both strategies in a single MIX model. We show that MIX significantly outperforms its preceding normalization methods. To validate the results, we use MIX to characterize caffeine metabolization measured in the finger sweat as well as diphenhydramine metabolization measured in blood plasma.

## Theory

### Probabilistic quotient normalization

*Definition.* Probabilistic quotient normalization (PQN) assumes that for a large, untargeted set of metabolites the median metabolite concentration fold change between two samples (e.g., two measured time points, $t_r$ and $t_s$) is approximately 1,

3a $$\begin{aligned} Q^\mathbf {C} = {{\,\mathrm{median}\,}}\left\{ \frac{C_j(t_r)}{C_j(t_{s})}\right\} \approx 1,\quad j\in [1,{n_{\text {metabolites}}}]. \end{aligned}$$

Consequently, fold changes calculated from $\widetilde{\mathbf {M}}$ instead of $\mathbf {C}$ are proportional to the ratio of $V$,

3b $$\begin{aligned} Q^\mathbf {M}&= Q^\mathbf {C} ~\frac{V(t_r)}{V(t_{s})} \approx \frac{V(t_r)}{V(t_{s})} \end{aligned}$$

with

3c $$\begin{aligned} Q^\mathbf {M}&= {{\,\mathrm{median}\,}}\left\{ \frac{\widetilde{M}_j(t_r)}{\widetilde{M}_j(t_s)}\right\} ,\quad j\in [1,{n_{\text {metabolites}}}]. \end{aligned}$$

In order to minimize the influence of experimental errors

4 $$\begin{aligned} M_j^\text {ref}= {{\,\mathrm{median}\,}}\left\{ \widetilde{M}_j(t_i)\right\} ,\quad i\in [1,n_{\text {time points}}] \end{aligned}$$

often replaces the dedicated sample in $\widetilde{M}_j(t_s)$ in the denominator of Eq. 3c [28]. Therefore, the normalization quotient by PQN is calculated as

5 $$\begin{aligned} Q^\text {PQN}(t)&= {{\,\mathrm{median}\,}}\left\{ \frac{\widetilde{M}_j(t)}{M_j^\text {ref}}\right\} ,\quad j\in [1,{n_{\text {metabolites}}}]. \end{aligned}$$

$Q^\text {PQN}$ is a relative measure and distributes around 1. In analogy to Eq. 3b, we define its relation to the (sweat) volume $V^\text {PQN}$ as

6 $$\begin{aligned} {\begin{matrix} Q^\text {PQN}(t)=\frac{V^\text {PQN}(t)}{V^{\text {ref}}}, \end{matrix}} \end{aligned}$$

where $V^{\text {ref}}$ denotes some unknown, time-independent reference (sweat) volume. Note that with real data only $Q^\text {PQN}(t)$ values can be calculated, but $V^\text {PQN}(t)$ as well as $V^{\text {ref}}$ remain unknown.

*Discussion.* $M_j^\text {ref}$ can be defined differently depending on the underlying data. However, the choice of reference is usually not critical to the outcome of PQN [28]. As no control or blank measurements are available, and the abundances of metabolites can range several orders of magnitudes, in this study, we used a metabolite-wise median reference for $Q^\text {PQN}$ calculation. Moreover, PQN might be sensitive to missing values; however, in this study, we only focused on (real and synthetic) data sets where 100% of values were present.

The biggest advantage of PQN is that no calibration curves and prior knowledge about changes over time of measured metabolites are required. Moreover, PQN is independent of the number of sample points measured in a time series. However, its major drawback is that the normalization quotient is not an absolute quantification and only shows relative changes. I.e., it does not quantify $V$ as given in Eq. 2 directly with an absolute value but instead normalizes relative abundances between samples and time points. Another critical assumption is that sweat metabolite concentrations need to be – on average – constant over the sampled time series. Whereas this is reasonable to assume for the sweat of healthy humans [20], one has to take care when investigating disease states (for example, cystic fibrosis, which is known to alter the sweat’s composition [31]).

### Pharmacokinetic normalization

*Definition.* In the pharmacokinetic model (PKM) we assume that we know at least the functional dependence, i.e. the pharmacokinetics, but not necessarily the value of the *k* (pharmaco-)kinetic parameters $\varvec{\uptheta }\in {\mathbb R}^k$ for $2\le \ell \le {n_{\text {metabolites}}}$ metabolites. Without loss of generality we (re-)sort $\widetilde{\mathbf {M}}$ such that the first $\ell$ elements (collected in the vector $\widetilde{\mathbf {M}}_\ell$) correspond to metabolites with known pharmacokinetic dependence, while the remaining ${n_{\text {metabolites}}}- \ell$ elements (collected in the vector $\widetilde{\mathbf {M}}_{\ell +}$) correspond to metabolites with unknown kinetics. Then Eq. 2 takes the form of

7a $$\begin{aligned} \begin{pmatrix} \widetilde{\mathbf {M}}_{\ell }\phantom{+} (t) \\ \widetilde{\mathbf {M}}_{\ell +}(t) \end{pmatrix}&=\begin{pmatrix}\mathbf {C}_{\ell}\phantom{+} (t;\varvec{\uptheta }) \\ \mathbf {C}_{\ell +}(t)\phantom{;\varvec{\uptheta}} \end{pmatrix} V^\text {PKM}(t) \end{aligned}$$

with physically meaningful bounds;

7b $$\begin{aligned} V_\text {lower bound}&\le V^\text {PKM}(t) \le V_\text {upper bound},\end{aligned}$$

7c $$\begin{aligned} \varvec{\uptheta }_\text {lower bound}&\le \quad~\varvec{\uptheta }~\quad \le \varvec{\uptheta }_\text {upper bound}. \end{aligned}$$

$V^\text {PKM}(t)$ as well as $\varvec{\uptheta }$ can be obtained by parametric fitting of $\widetilde{\mathbf {M}}_\ell ^\text {PKM}(t)$. Note that this allows not only to compute absolute values of $\mathbf {C}_\ell ^\text {PKM}(t;\varvec{\uptheta })$ but – with $V^\text {PKM}(t)$ – also of all other concentrations via $\mathbf {C}_{\ell +}(t)=\widetilde{\mathbf {M}}_{\ell +}(t)/V^\text {PKM}(t)$.

As $V^\text {PKM}(t_i)$ may be different at every time step $t_i$, we need to know the (pharmaco-)kinetics of at least two metabolites; otherwise, the number of parameters is larger than the number of data points.

*Discussion.* The biggest advantage of this method is that it can implicitly estimate absolute values of $V$ without the need for direct measurements. Therefore, sweat volumes can become smaller than the minimum required in volumetric methods, and shorter sampling times also become possible. A drawback of this method is the fact that it is only feasible if one has prior knowledge of relevant pharmacological parameters (i.e., ingested dose of metabolites of interest, volume of distribution, body mass of specimen, range of expected kinetic constants), which is limiting the approach to studies where at least two metabolites together with their pharmacokinetics are well known. Moreover, calibration curves of metabolites of interest and sufficiently many samples in a time series are required for robustly fitting the equation system. In a previously performed sensitivity analysis, an increase in the quality of fit was observed as the number of samples increased from 15 to 20 time points per measured time series [20].

### Mixed normalization

*Definition.* The mixed normalization model (MIX) is a combination of PQN and PKM. It is designed to incorporate robust statistics of untargeted metabolomics via its PQN term as well as an absolute estimation of $V$ via its PKM term.

Optimal parameters of MIX are found via optimization of two equations,

8a $$\begin{aligned} T \left[ \begin{pmatrix} \widetilde{\mathbf {M}}_{\ell }\quad (t) \\ \widetilde{\mathbf {M}}_{\ell +}(t) \end{pmatrix}\right]&= T\left[ \begin{pmatrix}\mathbf {C}_{\ell }\quad (t;\varvec{\uptheta }) \\ \mathbf {C}_{\ell +}(t)\quad \end{pmatrix} V^\text {MIX}(t)\right] \end{aligned}$$

and

8b $$\begin{aligned} ZT \left[ \mathbf {Q}^\text {PQN}(t)\right]&= ZT\left[ \mathbf {V}^\text {MIX}(t)\right] \end{aligned}$$

where additional transformations *T* (PKM and PQN term) and scaling *Z* (PQN term) can be applied to account for random and systematic errors (section "Hyperparameters") and $V^\text {MIX}(t)$ and $\varvec{\uptheta }$ are constrained between physically meaningful bounds,

8c $$\begin{aligned} V_\text {lower bound}&\le V^\text {MIX}(t) \le V_\text {upper bound}, \end{aligned}$$

8d $$\begin{aligned} \varvec{\uptheta }_\text {lower bound}&\le \quad ~\varvec{\uptheta }~\quad \le \varvec{\uptheta }_\text {upper bound}. \end{aligned}$$

E.g. bounds for $V$ can be calculated by Eq. 2b and minimal and maximal sweat rates from literature.

*Discussion.* We hypothesize that the MIX model can combine the advantages of PQN and PKM normalization models. Moreover, we believe that MIX inherits the statistical robustness of PQN while simultaneously estimating absolute values as fitted by PKM. Several prerequisites are necessary for normalization with PKM or MIX. However, if they are fulfilled, the improved goodness of normalization by using MIX instead of PKM usually does not come with an additional price as in many metabolomics studies, targeted and untargeted metabolites are measured in combination, and thus, all additional data required by MIX is already available.

## Methods

### Implementation

A generalized version of PKM and MIX (where an arbitrary number of independent metabolite kinetics can be modeled) was implemented as a Python class. As input it requires the number of metabolites used for kinetic modeling ($\ell$), a vector of time points as well as the measured mass data ($\widetilde{\mathbf {M}}$, matrix with time points in the rows and metabolites in the columns). MIX additionally takes a $\mathbf {Q}^\text {PQN}= [Q^\text {PQN}(t_1),...,Q^\text {PQN}(t_{n_{\text {time points}}})]^\text {T}$ vector (calculated with the PQN method from all metabolites, ${n_{\text {metabolites}}}$) for all time points of a time series. Upon optimization (carried out with self.optimize_monte_carlo, which is a wrapper for SciPy’s optimize.curve_fit [32]) the kinetic constants and sweat volumes are optimized to the measured data by minimizing the functions listed in Eqs. 9b and 9c for PKM and MIX respectively:

9a $$\begin{aligned} \text {min}(\mathcal {L}^\text {MIX})&= \text {min}(\mathcal {L}^\text {PKM}+ \mathcal {L}^\text {PQN}) \end{aligned}$$

where

9b $$\begin{aligned} \mathcal {L}^\text {PKM}&= \sum ^{n_\text {time points}}_{i=1} \sum ^{n_\text {metabolites}}_{j=1} L\left[ \lambda \left( T(\widetilde{M}_{ij}) - T(C_{ij}~V^\text {MIX}_{i})\right) ^2\right] , \end{aligned}$$

9c $$\begin{aligned} \mathcal {L}^\text {PQN}&= \sum ^{n_\text {time points}}_{i=1} L\left[ (1-\lambda )\left( ZT(\mathbf {V}^\text {MIX})_i-ZT(\mathbf {Q}^\text {PQN})_i\right) ^2 \text {Var}(T(\mathbf {V}^\text {MIX})) \right] , \end{aligned}$$

$\text {Var}(\mathbf {V})$ is the variance of $\mathbf {V}$ (which is the vector of estimated *V* over all time points), *T* is a transformation function, *Z* is a scaling function, and *L* is the loss function. The key difference between PKM and MIX is that the fitted $V$ in MIX are biased towards relative abundances as calculated by PQN. An important additional hyperparameter of the MIX model is $\lambda$, which weights the error residuals of $\mathcal {L}^\text {PKM}$ and $\mathcal {L}^\text {PQN}$. Its calculation is discussed in section "Hyperparameters". If $\lambda = 1$, the MIX model simplifies again to a pure PKM model.

**Fig. 1:**
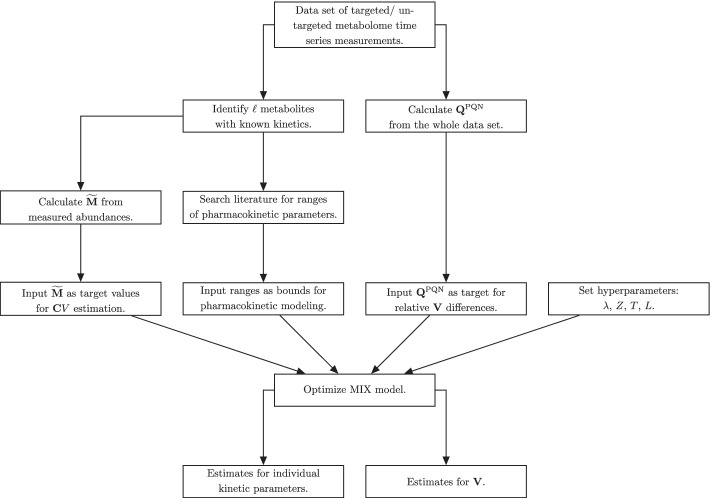
Flow chart for data processing for MIX normalization

To summarize, an overview of the differences between PKM and MIX models is given in Additional file 1: Table S1 and a flow chart of data processing for MIX normalization is given in Fig. 1.

**Fig. 2:**
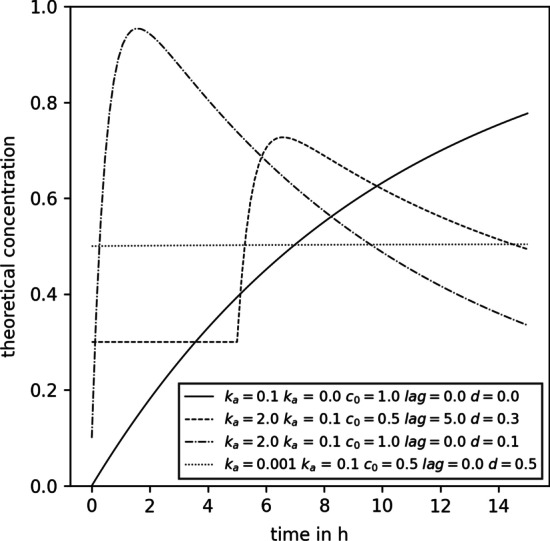
Examples of concentration time series that can be modeled with the modified Bateman equation used. The legend shows the kinetic parameters used to create the respective curves. All parameters are within the bounds that were used for kinetic parameter fitting

#### Hyperparameters

Several hyperparameters can be set for the PKM and MIX Python classes.

*Kinetic function.* Firstly, it is possible to choose the kinetic function used to calculate $\mathbf {C}$. In this study we focused on a modified Bateman function *F*(*t*) with 5 kinetic parameters ($k_a,~k_e,~c_0,~lag,~d$):

10a $$\begin{aligned} F(t) = \left\{ \begin{array}{ll} b(t) + d &{} \text{ if } b(t) \ge 0 \\ d &{} \text{ if } b(t) < 0 \end{array} \right. \end{aligned}$$

with

10b $$\begin{aligned} b(t) = c_0~\frac{k_a}{k_e-k_a}~\left( e^{-k_a(t-lag)} - e^{-k_e(t-lag)}\right) . \end{aligned}$$

This function was designed to be flexible and able to represent several different metabolite consumption and production kinetics, as exemplified by Fig. 2. Intuitively, $k_a$ and $k_e$ correspond to kinetic constants of absorption and elimination of a metabolite of interest with the unit h$^{-1}$. $c_0$ is the total amount of a metabolite absorbed over the volume of distribution with the unit mol L$^{-1}$. Additionally to these parameters which are also part of the classical Batman function [33], we here introduce *lag* and *d*. The *lag* term with the unit h shifts the function along the X-axis, intuitively defining the starting time point of absorption of a metabolite of interest, whereas the *d* term with the unit mol L$^{-1}$ shifts the function along the Y-axis.

*Loss function*, *L*. *L* calculates the loss value after estimation of the error residuals of the model (Eq. 9). It can be set via self.set_loss_function to either cauchy_loss or max_cauchy_loss (or max_linear_loss). In both cases the loss is calculated as a Cauchy distribution of error residuals according to SciPy [32]. The difference, however, is that cauchy_loss only uses the absolute error residuals, whereas max_cauchy_loss uses the maximum of relative and absolute error residuals (thus the word max is expressed in its name). The reason for its addition was that a good performance has been achieved in a previous study [20]. In this study we used the max_cauchy_loss loss function for PKM models and cauchy_loss for MIX models. The choice of *L* is intertwined with the choice of *T* which becomes clear in the following paragraph.

*Transformation function*, *T*. *T* transforms the measured data $\widetilde{\mathbf {M}}$ as well as the calculated $\mathbf {Q}^\text {PQN}$, $\mathbf {C}V$, and $\mathbf {V}$ before calculation of the loss (Eq. 9). Two different transformations, none and log10, can be set during initialization with the argument trans_fun. As originally reported [20] no transformation was done for PKM (i.e. trans_fun=’none’),

11a $$\begin{aligned} T(\widetilde{\mathbf {M}}) = \widetilde{\mathbf {M}}. \end{aligned}$$

For MIX models, however, a log-transform was performed (i.e. trans_fun=’log10’),

11b $$\begin{aligned} T(\widetilde{\mathbf {M}}) = \log _{10}(\widetilde{\mathbf {M}}+ 10^{-8}) \end{aligned}$$

as the error on measured data is considered multiplicative [34] and the sweat volume log-normally distributed (Additional file 1: Fig. S1). To avoid problems with concentrations of the size 0 a small number (i.e., the size of optimizer precision [32]) is added.

In a sensitivity analysis study, we tested the quality of normalization of MIX with different *L* and *T* hyperparameters and concluded that a combination of cauchy_loss for *L* and log10 for *T* performed best (Additional file 1: Fig. S2C, D). This is in agreement with literature where logarithmic transformations performed well in combination with PQN for size effect normalization of sweat measurements [35].

*Scaling function*, *Z*. *Z* describes a scaling function performed on $T(\mathbf {Q}^\text {PQN})$ and $T(\mathbf {V})$. Scaling is performed to correct for noisy data (see Results section "In fluence of noise on PQN"). Two strategies can be set with the scale_fun argument during initialization of the MIX model class, standard or mean. In this study, all MIX models employ standard scaling, i. e.

12a $$\begin{aligned} ZT(\mathbf {Q}^\text {PQN})&= \frac{T(\mathbf {Q}^\text {PQN}) - \text {mean}(T(\mathbf {Q}^\text {PQN}))}{\text {Std}(T(\mathbf {Q}^\text {PQN}))}. \end{aligned}$$

We additionally implemented mean scaling which differs depending on the choice of *T* with

12b $$\begin{aligned} ZT(\mathbf {Q}^\text {PQN})&= \left\{ \begin{array}{ll} T(\mathbf {Q}^\text {PQN}) - \text {mean}(T(\mathbf {Q}^\text {PQN})) &{} \text{ if } \texttt {trans\_fun='log10'} \\ T(\mathbf {Q}^\text {PQN}) / \text {mean}(T(\mathbf {Q}^\text {PQN})) &{} \text{ if } \texttt {trans\_fun='none'}. \end{array} \right. \end{aligned}$$

*Optimization strategy.* The optimization of both PKM and MIX models is done with a Monte Carlo strategy where the initial parameters are sampled randomly from a uniform distribution between their bounds. Performing a sensitivity analysis, we previously showed that this method is preferable to a single fitting procedure [20]. In this study, the number of Monte Carlo replicates for model fitting was set to 100.

*Weighting of MIX loss terms.* A weighting constant for every measured data point can be used by the model. In a sensitivity analysis study, we found that the choice of $\lambda$ is not critical to the quality of normalization as long as it is not extremely tilted to one side (i.e., $\lambda$ close to 0 or 1, Additional file 1: Fig. S2A, B). Thus we propose a method where the loss terms are weighted by the number of data points fitted for each of both loss terms but not by the number of metabolites used in the calculation of each term (Additional file 1: Equation S1). For such a method the solution for $\lambda$ is given by Eq. 13.

13 $$\begin{aligned} \lambda = \frac{1}{\ell + 1} \end{aligned}$$

#### Full and minimal models

In this study, we differentiate between full and minimal models. With full models, we refer to pharmacokinetic normalization models (PKM or MIX) where all metabolites of a given data set are used for the pharmacokinetic normalization. This means that, for example, if ${n_{\text {metabolites}}}= 20$ all 20 metabolites were modeled with the modified Bateman function and thus in Eqs. 7a and 8a, $\ell = {n_{\text {metabolites}}}$ and $\widetilde{\mathbf {M}}_{\ell +}$ is an empty vector. On the other hand, minimal models are models where only the few known, better constrained metabolites were modeled with a kinetic function. This means that the information used for PKM$_{\text {minimal}}$   does not change upon the addition of (synthetic) metabolites. Therefore, its goodness of fit measure should stay constant within statistical variability upon change of ${n_{\text {metabolites}}}$. This behaviour was used to verify if the simulations worked as intended and if no biases in the random number generation existed. On the other hand, the MIX$_{\text {minimal}}$ model still gained information from the increase of ${n_{\text {metabolites}}}$ as the PQN part of this model was calculated with all ${n_{\text {metabolites}}}$. Therefore, changes in the goodness of fit measures for MIX$_{\text {minimal}}$ are expected. We emphasize that the definition of full and minimal models is specific to this particular study. Here we explicitly set $\ell = 4$, which originates from previous work where 4 targeted metabolites (caffeine, paraxanthine, theobromine, theophylline) with known kinetics were measured [20].

**Fig. 3:**
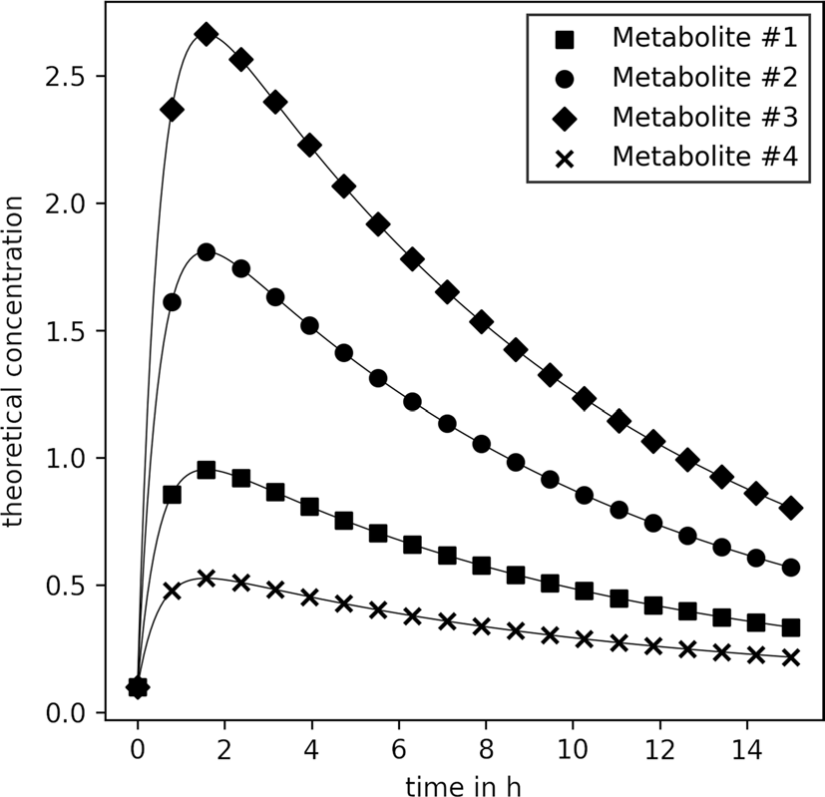
Theoretical concentration $\mathbf {C}$ for the first four metabolites of the synthetic data. Kinetic parameters used for calculation are listed in Additional file 1: Table S2

### Synthetic data creation

Three different types of synthetic data sets were investigated. The first two types of data sets (sampled from kinetics, section "Sampled kinetics" and sampled from means and standard deviations, section "Sampled mean and standard deviation") test the behaviour of normalization models in extreme cases (either all metabolites describable by pharmacokinetics or all metabolites completely random). Finally, the third type of data set (sampled from real data, section "Sampled from real data") aims to replicate measured finger sweat data as close as possible. In sum, the performance of normalization methods on all three types of data sets can show how they behave in different situations with different amounts of describable data.

In all three cases, data creation started with a simple toy model closely resembling the concentration time series of caffeine and its degradation products (paraxanthine, theobromine, and theophylline) in the finger sweat as described elsewhere [20]. The respective parameters are listed in Additional file 1: Table S2. With them, the concentration of metabolites #1 to #4 were calculated for 20 time points (between 0 and 15 h in equidistant intervals, Fig. 3). Subsequently, new synthetic metabolite concentration time series were sampled and appended to the toy model (i.e., to the concentration vector, $\mathbf {C}(t)$). Three different synthetic data sampling strategies were tested, and their specific details are explained in the following sections. Next, sweat volumes ($V$) were sampled from a log-normal distribution truncated at ($0.05 \le V\le {4}\,\upmu \hbox {L}$) closely resembling the distribution of sweat volumes estimated in our previous publication [20], Additional file 1: Fig. S1. Finally, an experimental error ($\varvec{\upepsilon }$) was sampled for every metabolite and time point from a normal distribution with a coefficient of variation of $20\%$ and the synthetic data was calculated as

14 $$\begin{aligned} \widetilde{\mathbf {M}}(t) = \text {diag}\big (\mathbf {C}(t)~V(t)\big )~\varvec{\upepsilon }(t)\end{aligned}.$$

For every tested condition, 100 synthetic data replicates were generated, and the normalization models were fitted.

#### Sampled kinetics

In simulation v1, data was generated by sampling kinetic parameters for new metabolites from an uniform distribution. The distribution was constrained by the same bounds also used for the PKM and MIX model fitting: $(0,0,0,0)^\text {T} \le (k_a,k_e,c_0,lag,d)^\text {T} \le (3,3,5,15,3)^\text {T}$. Subsequently the concentration time series of the synthetic metabolites were calculated according to the modified Bateman function (Eq. 10).

#### Sampled mean and standard deviation

Means and standard deviations of the concentration time series of metabolites were calculated from untargeted real finger sweat data (for details, see section "Real finger sweat metabolome data"). The probability density function of both can be described by a log-normal distribution (Additional file 1: Fig. S3). For the data generation of simulation v2, per added metabolite, one mean and one standard deviation were sampled from the fitted distribution and used as an input for another log-normal distribution from which a random concentration time series was subsequently sampled. This results in synthetic concentration values that behave randomly and, therefore, cannot be easily described by our pharmacokinetic models.

#### Sampled from real data

To get an even better approximation to real data, in simulation v3, concentration time series were directly sampled from untargeted real finger sweat data (for details, see section "Real finger sweat metabolome data"). To do so, the untargeted metabolite $\widetilde{\mathbf {M}}$ time series data set was normalized with PQN. As the number of metabolites in this data set was comparably large (${n_{\text {metabolites}}}= 3446$) we could assume that the relative error (or rRMSE, for more explanation, see section "Synthetic data simulations") was negligibly small. The resulting values are, strictly speaking, fractions of concentrations. However, this does not affect the results as these values are anyways considered untargeted (i.e., no calibration curve exists) and thus relative. Therefore, the PQ normalized data set could be used as ground truth for concentration time series sampling. Subsequently, a subset of the original ground truth data was sampled for synthetic data generation.

#### Sampling of noisy data

We investigated the influence of background (i.e. noisy) signal on the performance on $\mathbf {Q}^\text {PQN}$ (and scaled and transformed variants thereof). To simulate such an environment we used data sampled from real data (section "Sampled from real data"), and applied $V$ only to a fraction of the $\mathbf {C}$ vector,

15 $$\begin{aligned} \begin{pmatrix} \widetilde{\mathbf {M}}\phantom{n}(t) \\ \widetilde{\mathbf {M}}_{n}(t) \end{pmatrix}&= \text {diag} \begin{pmatrix} \mathbf {C}\phantom{n}(t)V(t) \\ \mathbf {C}_{n}(t)\phantom{V(t)} \end{pmatrix} ~\varvec{\upepsilon }. \end{aligned}$$

The noise fraction is given by the number of elements of $\widetilde{\mathbf {M}}$ and $\widetilde{\mathbf {M}}_n$ vectors,

16 $$\begin{aligned} f_n = \frac{\text {length} (\widetilde{\mathbf {M}}_n)}{\text {length} (\widetilde{\mathbf {M}}) + \text {length}( \widetilde{\mathbf {M}}_n)}, \end{aligned}$$

where subscript *n* in $\widetilde{\mathbf {M}}_n, \mathbf {C}_n,$ and $f_n$ denotes them as part of the noise.

Simulations were carried out for 20 equidistant noise fractions between $0 \le f_n \le 0.95$ with ${n_{\text {metabolites}}}= 100$ and $n_{\text {time points}}= 20$ for 100 replicates. The error residuals of mean and standard scaled $\mathbf {Q}^\text {PQN}$ are calculated as

17a $$\begin{aligned} \text {Mean Scaled Error}&= \sum _i^{n_{\text {time points}}} \left[ ZT(\mathbf {Q}^\text {PQN})_i - ZT(\mathbf {V})_i\right] \end{aligned}$$

with *Z* defined as in Eq. 12b and

17b $$\begin{aligned} \text {Standard Scaled Error}&= \sum _i^{n_{\text {time points}}} \left[ ZT(\mathbf {Q}^\text {PQN})_i - ZT(\mathbf {V})_i \right] \text {Std}(T(\mathbf {V})) \end{aligned}$$

with *Z* defined as in Eq. 12a. For both cases *T* is defined as the logarithm (Eq. 11b). We point out that the multiplication with $\text {Std}(T(\mathbf {V}))$ for the standard scaled error is important to make the results comparable, as otherwise the error would be biased towards the method with smaller scaled standard deviation regardless of the performance of the scaling.

### Normalization model optimization

Normalizing for the sweat volume by fitting kinetics through the measured values only has a clear advantage over PQN if it is possible to infer absolute sweat volumes and concentration data. In order to be able to do that, some information about the kinetics and the starting concentrations of metabolites of interest need to be known. For example, when modeling the caffeine network in our previous publication [20], we knew that the *lag* parameter of all metabolites was 0 and that the total amount of caffeine ingested (which corresponds to $c_0$) was 200 mg. Moreover, we knew that caffeine and its metabolites are not synthesized by humans and implemented the same strategy into our toy model (corresponding to *d*). As the toy model was designed to resemble such a metabolism, we translated this information to the current study. Therefore, we assumed that the first 4 metabolites in our toy model had known $c_0$, *lag*, and *d* parameters. For their corresponding $k_a$ and $k_e$ and the parameters of all other metabolites the bounds were set to the same $(0,0,0,0)^\text {T} \le (k_a,k_e,c_0,lag,d)^\text {T} \le (3,3,5,15,3)^\text {T}$ used in kinetic data generation. Fig. 2 shows examples of concentration time series that can be described with the modified Bateman function and parameters within the fitting bounds.

### Real finger sweat metabolome data

The real world finger sweat data was extracted from 37 time series measurements of Study C from ref. [20]. It was downloaded from MetaboLights (MTBLS2772 and MTBLS2776).

*Preprocessing.* The metabolome data set was split into two parts: targeted and untargeted. The targeted data (i.e., the mass time series data for caffeine, paraxanthine, theobromine, and theophylline) was directly adopted from the mathematical model developed by [36]. This data is available on GitHub (https://github.com/Gotsmy/finger_sweat).

For the untargeted metabolomics part, the raw data was converted to the mzML format with the msConvert tool of ProteoWizard (version 3.0.19228-a2fc6eda4) [37]. Subsequently, the untargeted detection of metabolites and compounds in the samples was carried out with MS-DIAL (version 4.70) [38]. A manual retention time correction was first applied with several compounds present in the majority (more than 90%) of the samples. These compounds were single chromatographic peaks with no isomeric compounds present at earlier or later retention times (*m*/*z* 697.755 at 5.57 min, *m*/*z* 564.359 at 5.10 min, *m*/*z* 520.330 at 4.85 min, *m*/*z* 476.307 at 4.58 min, *m*/*z* 415.253 at 4.28 min, *m*/*z* 371.227 at 3.95 min, *m*/*z* 327.201 at 3.56 min, *m*/*z* 283.175 at 3.13 min, *m*/*z* 239.149 at 3.63 min, *m*/*z* 166.080 at 1.69 min, *m*/*z* 159.113 at 1.19). After this, untargeted peak detection and automated alignment (after the manual alignment) were carried out with the following settings: Mass accuracy MS1 tolerance: 0.005 Da, Mass accuracy MS2 tolerance: 0.025 Da, Retention time begin: 0.5 min, Retention time end: 6 min, Execute retention time correction: yes, Minimum peak height: 1E5, Mass slice width: 0.01 Da, Smoothing method: Linear weighted moving average, Smoothing level: 3 scans, Minimum peak width: 5 scans, Alignment reference file: C_D1_I_o_pos_ms1_1.mzML, Retention time tolerance: 0.3 min, MS1 tolerance: 0.015 Da, Blank removal factor: 5 fold change). No blank-subtraction was carried out as the internal standard caffeine was spiked into each sample, including the blanks. Peak abundances and meta-information were exported with the Alignment results export functionality.

Subsequently, we excluded isomers within a *m/z* difference of less than ${0.001}\,\hbox {Da}$ and a retention time difference of less than ${0.5}\,\hbox {min}$. To further reduce features that are potentially background, features with retention times after ${5.5}\,\hbox {min}$ as well as features with minimal sample abundances of $< 5\times \text {maximum blank abundance}$ (except for the internal standard, caffeine-D9) were excluded from the data set. This was done on a time series-wise basis. Thus the number of untargeted metabolites considered for normalization differs with a mean of $343\pm 152$ for the 37 time series of interest.

**Fig. 4:**
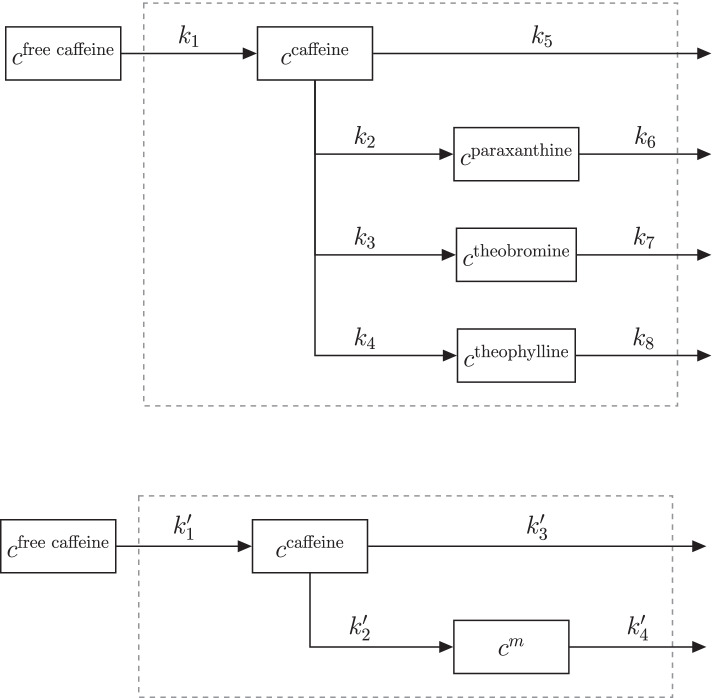
Full network (top panel) and subnetwork (bottom panel) of caffeine absorption, conversion to paraxanthine, theobromine, and theophylline and their elimination. The system boundary (dashed line) represents the human body. $m \in \{\text {paraxanthine, theobromine, and theophylline}\}$

*Size effect normalization.* In this finger sweat data set, time series of targeted as well as untargeted metabolomics, are listed. The kinetics of the four targeted metabolites (caffeine, paraxanthine, theobromine, and theophylline) are known. A reaction network of the metabolites is shown in the top panel of Fig. 4. Briefly, caffeine is first absorbed and then converted into three degradation metabolites. Additionally, all four metabolites are eliminated from the body. All kinetics can be described with first order mass action kinetics [39, 40].

In order to assess the performance of the sweat volume normalization methods, the full network was split up into three subnetworks that all contained caffeine and one degradation metabolite each (Fig. 4 bottom panel). The solution of the first order differential equations describing such network is given in Additional file 1: Eqs. S2a and S2b. Moreover, the $343\pm 152$ untargeted metabolite time series were randomly split up into three (almost) equally sized batches, and each batch was assigned to one subnetwork. All three networks were subsequently separately normalized with PKM$_{\text {minimal}}$   and MIX$_{\text {minimal}}$ methods with kinetic parameters that were adjusted to the specific reaction network (Fig. 4 bottom panel). Subsequently, the kinetic constants ($k_1'$, $k_2'$, $k_3'$, $k_4'$) were estimated for 37 measured concentration time series. Fitting bounds were not changed in comparison to the original publication [20].

As all three subnetwork data sets originate from the same finger sweat measurements, the underlying kinetic constants should be exactly identical. As the kinetic constants of absorption ($k_a^\text {caf}= k_1'$) and elimination ($k_e^\text {caf}= k_2' + k_3'$) of caffeine are estimated in all three subnetworks, we used their standard deviation to test the robustness of the tested normalization methods.

### Real blood plasma metabolome data

In the study of Panitchpakdi et al. [41] the mass time series of the metabolome was measured in different body fluids after the uptake of diphenhydramine (DPH). Here, we focus on data measured in the blood plasma, which includes the abundances of DPH (known kinetics, calibration curve, pharmacological constants) as well as three of its metabolization products (known kinetics) and the abundances of 13526 untargeted metabolites with unknown kinetics.

*Preprocessing.* The data of peak areas was downloaded from the GNPS platform [42]. To reduce the number of metabolites that are potentially background and/or noise in the data set, features with minimal sample abundances of $< 5\times \text {maximum blank abundance}$ were excluded from the data set on a time series-wise basis. Thus, the number of untargeted metabolites considered for normalization differs with a mean of $1017\pm 114$ for the 10 time series of interest.

*Size effect normalization.* We assume that the kinetics of four metabolites (DPH, N-desmethyl-DPH, DPH N-glucuronide, and DPH N-glucose) can be described by the modified Bateman (Eq. 10). A reaction network of the metabolites is shown in Additional file 1: Fig. S4. Briefly, DPH is first absorbed and then – with unknown intermediates – converted into three degradation metabolites, which are in turn metabolized further downstream or eliminated. $c_0$ of DPH was calculated with pharmacological constants for bioavailability, volume of distribution, and dosage of DPH as reported in the original publication [41].

Analogously to the normalization performed on finger sweat data, the full network of four metabolites is split up into three subnetworks with only one, shared, targeted metabolite (DPH itself), one additional untargeted metabolite with known kinetic (either N-desmethyl-DPH, DPH N-glucuronide, or DPH N-glucose, Additional file 1: Fig. S5) and one third of $1017\pm 114$ untargeted metabolites with unknown kinetics. To ensure better convergence during fitting of the models, the $\widetilde{\mathbf {M}}$ data was first scaled to values between 0 and 1 by dividing by its metabolite-wise maximum. This factor can be multiplied again as part of $c_0$ after the normalization is done. Thereafter, PKM$_{\text {minimal}}$ and MIX$_{\text {minimal}}$ models were fitted onto the scaled $\widetilde{\mathbf {M}}$ data (with $\ell = 2$) for all ten measured time series. The bounds of parameters were chosen so that previously reported estimates [41] are well within range: $0 \le k \le {5}\,\hbox {h}^{-1}$ for $\{k_1', k_3'\}$, $0 \le k \le {1}\,\hbox {h}^{-1}$ for $\{k_2', k_4'\}$, $c_0^\text {DPH}$ as reported in the original publication normalized by the maximum factor, $0\le c_0^m\le 300$ for $m \in \{$N-desmethyl-DPH, DPH N-glucuronide, DPH N-glucose$\}$ and $lag = d = 0$ as well as $0.01 \le V\le {0.03}\,\hbox {mL}$.

As all three subnetwork data sets originate from the same plasma time series measurements, the underlying kinetic constants of DPH should be exactly identical. As the kinetic constants of absorption ($k_a^\text {DPH}= k_1'$) and elimination ($k_e^\text {DPH}= k_2'$) of DPH are estimated in all three subnetworks we used their standard deviation to test the robustness of PKM$_{\text {minimal}}$ and MIX$_{\text {minimal}}$.

### Data analysis

*Goodness of normalization.* Two goodness of fit measures were calculated to analyze the performance of the tested methods. RMSE is the standard deviation of the residuals of a sampled sweat volume time series vector ($\mathbf {V}^\text {true}$) minus the fitted sweat volume vector ($\mathbf {V}^\text {fit}$), while rRMSE is the standard deviation of the ratio of sampled and fitted $\mathbf {V}$ vectors normalized by its mean. Intuitively, RMSE is a measure of how much absolute difference there is between the fit and a true value, rRMSE, on the other hand, gives an estimate of how good the fitted sweat volumes are relative to each other. A visual depiction of RMSE and rRMSE is shown in Additional file 1: Fig. S6 and their exact definition is given in the equations in 3.3.

*Statistical analysis.* The significant differences in the mean of goodness of fit measures were investigated by calculating *p* values with the non-parametric pairwise Wilcoxon signed-rank test [43] (SciPy’s stats.wilcoxon function [32]). Significance levels are indicated by *, **, and *** for $p \le 0.05$, 0.01, and 0.001 respectively.

## Results

**Fig. 5:**
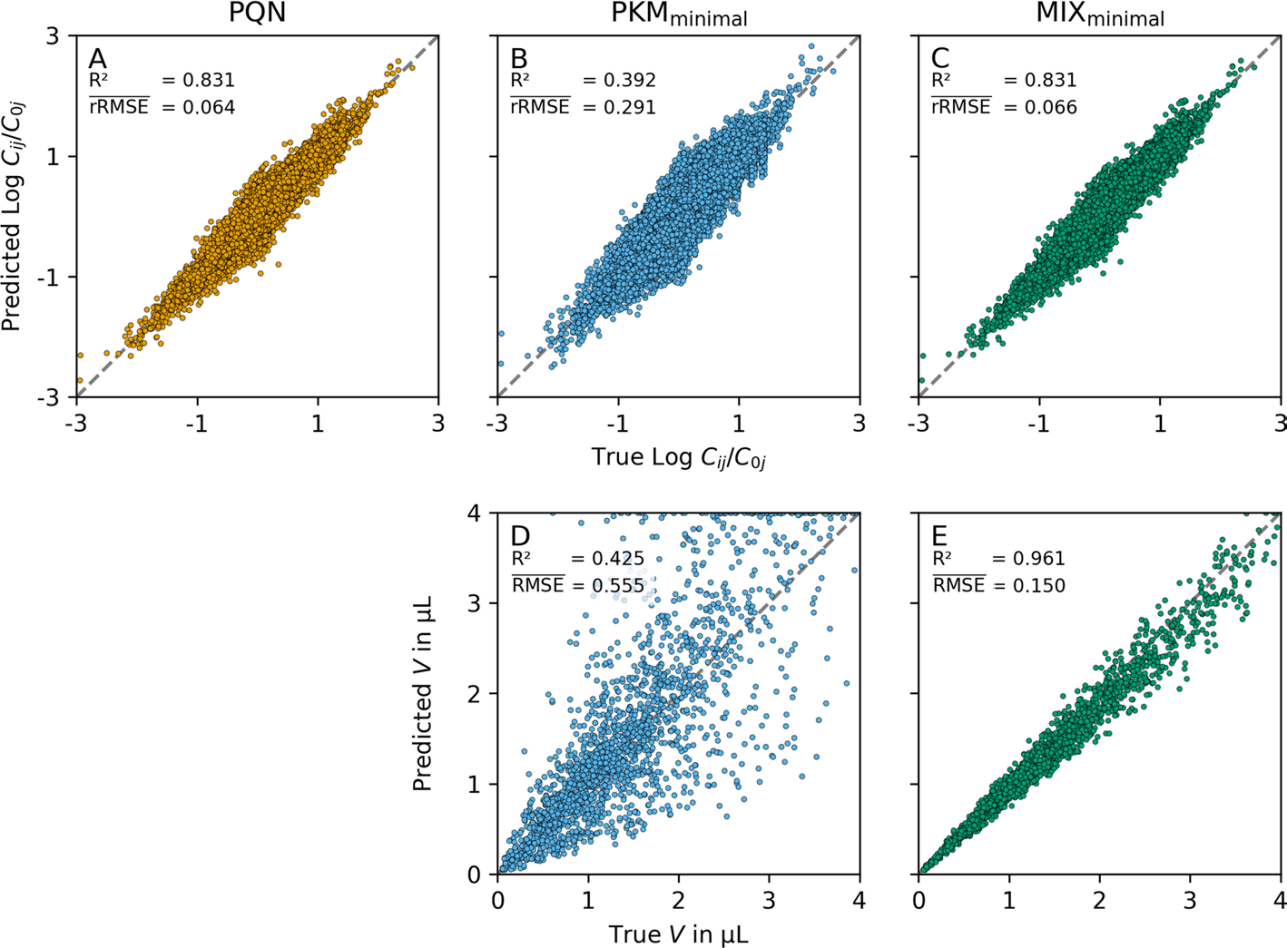
Relative and absolute normalization performance. In the top row the predicted $\text {log}_{10}(C_{j}(t_i;\varvec{\uptheta })/C_{j}(0;\varvec{\uptheta }))$ ($i \in \{1, ..., n_{\text {time points}}\}$, $j \in \{1, ..., {n_{\text {metabolites}}}\}$) are plotted as a function of the true, underlying $\text {log}_{10}(C_{j}(t_i)/C_{j}(0))$. The bottom row shows the predicted $V$ as a function of the true, underlying $V$. The columns represent different normalization models (PQN, PKM$_{\text {minimal}}$, and MIX$_{\text {minimal}}$ from left to right). As no absolute $V$ can be calculated from PQN the bottom left plot is omitted. To illustrate the effect of different RMSE and rRMSE sizes (which both are calculated from $V$), we show their mean over 100 replicates in comparison to the R$^2$  values calculated from the points plotted. Intuitively rRMSE is a measure of good correlation on the top row whereas RMSE is a measured of good correlation on the bottom row (high R$^2$, low rRMSE/RMSE respectively)

### Comparison of PKM and MIX

#### Synthetic data simulations

In order to test the performance of different normalization models, we generated 100 synthetic data sets with three different methods (simulations v1, v2, v3) and five different ${n_{\text {metabolites}}}$ (4, 10, 20, 40, 60) each, where the underlying $\mathbf {C}$, $V$, and $\varvec{\upepsilon }$ values were known. Simulations v1, v2, and v3 differ in the way how $\mathbf {C}$ was generated (kinetic, random, sampled from real data set, respectively). In order to quantify the normalization model performance, two measures of goodness of normalization were used for the analysis of the results: RMSE and rRMSE.

To visualize the obtained normalization performances we plotted the results for simulation v3 and ${n_{\text {metabolites}}}= 60$ in Fig. 5 for three normalization models (from left to right column, PQN, PKM$_{\text {minimal}}$, and MIX$_{\text {minimal}}$). The top row shows the predicted $\text {log}_{10}(C_{j}(t_i;\varvec{\uptheta })/C_{j}(0;\varvec{\uptheta }))$ (i.e. the concentration of each metabolite *j* at each time point *i* divided by its concentration at time 0) as a function of the true $\text {log}_{10}(C_{j}(t_i)/C_{j}(0))$ values. It illustrates the correlation of the relative abundances of one metabolite across all time points. Good correlations (i.e. high R$^2$) as seen for PQN and MIX$_{\text {minimal}}$ result in a low rRMSE measure. On the bottom row of Fig. 5 the absolute values of predicted $V$ are plotted as a function of the true $V$. There it becomes evident that good correlations of absolute values result in low RMSE measures.

In the following sections, we will focus on the size of RMSE and rRMSE, respectively, as they are both calculated from the predicted $V$ directly. Note that for PQN, no absolute $V$ can be estimated and, therefore, no RMSE is calculated.

**Fig. 6:**
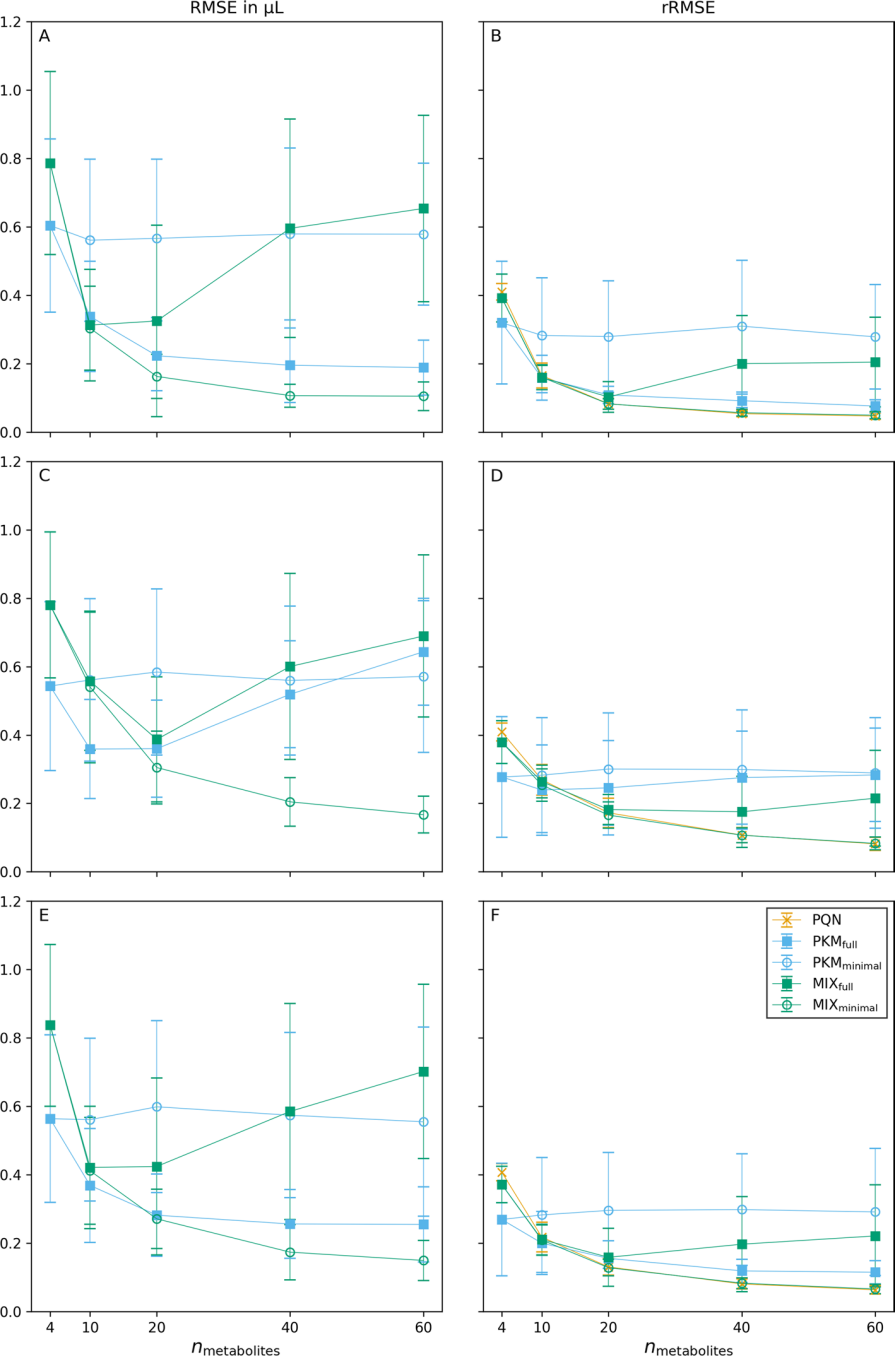
Goodness of normalization measures of synthetic data simulations. The mean for 100 replicates for different sweat volume normalization models is given for RMSE (left column) and rRMSE (right column). Results for simulations v1, v2, and v3 are shown in rows one, two, and three, respectively. The error bars represent standard deviations of the replicates. For the PQN method no RMSE can be calculated

*Influence of the number of metabolites.* We tracked RMSE and rRMSE of normalization methods for different numbers of metabolites (${n_{\text {metabolites}}}$) to investigate how the methods behave with different amounts of available information. An overview of their goodness of normalization measures as a function of ${n_{\text {metabolites}}}$ on sampled kinetic data (panels A, B), on completely random data (panels C, D), and on sampled subsets of real data (panels E, F) is given in Fig. 6.

PKM$_{\text {full}}$ which fits a kinetic function through all possible metabolites ($\ell = {n_{\text {metabolites}}}$) performs well (low RMSE, low rRMSE) when the $\mathbf {C}$ data originates from a kinetic function (simulation v1, Fig. 6A, B). However, when the underlying data does not originate from kinetic time series (simulation v2, Fig. 6C, D) its performance is reduced drastically. For PKM$_{\text {full}}$ this is resembled in an increase of RMSE (from $0.19 \pm {0.08}\,\upmu \hbox {L}$ to $0.64 \pm {0.16}\,\upmu \hbox {L}$ for ${n_{\text {metabolites}}}= 60$) as well as of rRMSE (from $0.08 \pm 0.02$ to $0.28 \pm 0.14$ for ${n_{\text {metabolites}}}= 60$).

Another observation is the behaviour of PQN. Its rRMSE approaches a value close to 0 with increasing ${n_{\text {metabolites}}}$, indifferently on how the underlying data was generated.

Interestingly, the results from simulation v3 lie between the results from simulations v1 and v2. This gets especially evident when comparing the performance of PKM$_{\text {full}}$ in Fig. 6. Such a result suggests that not all of the untargeted metabolites measured are completely random, but some can be described with the modified Bateman function. This leads to the hypothesis that after sweat volume normalization, the real finger sweat data (from which values for v3 were sampled) has a high potential for discoveringunknown kinetics.

Exact numbers for RMSE and rRMSE for all normalization methods and ${n_{\text {metabolites}}}$ are given in Additional file 1: Tables S3 and S4 respectively. Moreover, pairwise comparisons of RMSE and rRMSE of normalization methods relative to the results from PKM$_{\text {minimal}}$ are plotted in Additional file 1: Fig. S7.

**Fig. 7:**
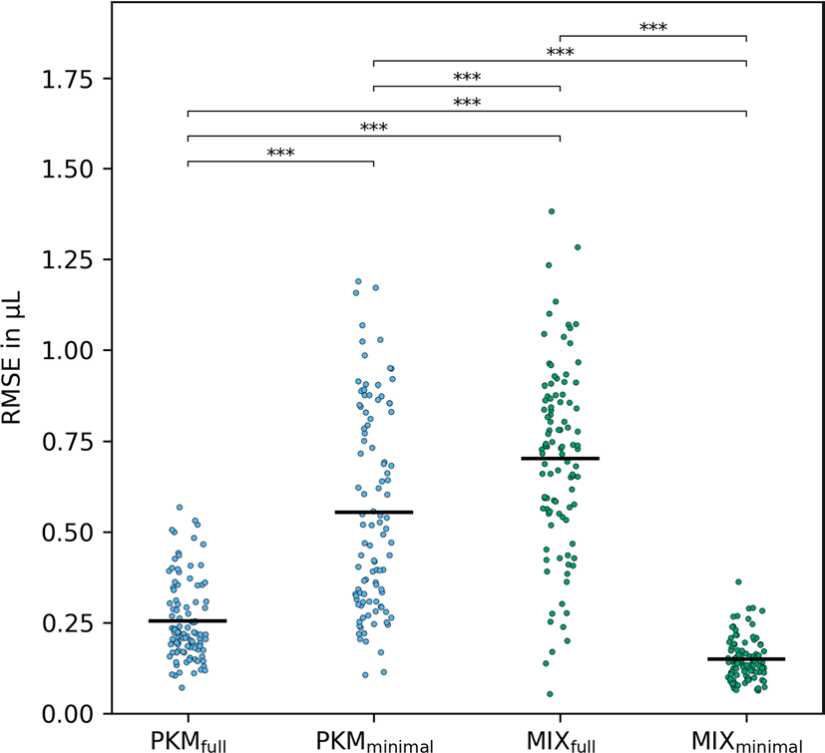
RMSE measures of simulation v3 with ${n_{\text {metabolites}}}= 60$. The significance between the methods was calculated on 100 paired replicates with the two-sided Wilcoxon signed-rank test

*Statistical testing.* As at ${n_{\text {metabolites}}}= 60$ the goodness of normalization measures started to flatten out, we further investigated this condition for statistical significance. We used the two-sided non-parametric Wilcoxon signed-rank test to compare pairwise differences in RMSE and rRMSE between the tested models. *p*-values for all combinations are given in Additional file 1: Tables S5 and S6.

As Fig. 6 already indicated, the overall best performance in RMSE as well as rRMSE is observed for the MIX$_{\text {minimal}}$ model. For ${n_{\text {metabolites}}}= 60$ it significantly outperforms every other method’s RMSE (Fig. 7). Moreover, MIX$_{\text {minimal}}$ ’s performance in rRMSE is at least equal to or better than all other tested methods (Additional file 1: Table S6) with one exception: the comparison of rRMSE of MIX$_{\text {minimal}}$ and PQN in simulation v1 shows significant difference ($p = 0.0029$), however, the absolute values of rRMSE are still very similar ($0.049\pm 0.010$ and $0.047\pm 0.009$ respectively). Compared to the previously used PKM$_{\text {minimal}}$ [20], the RMSE of MIX$_{\text {minimal}}$ improves by $73 \pm {10}\,\%$, the rRMSE by $43 \pm {12}\,\%$ (Additional file 1: Fig. S7). Analogously to Fig. 7 for simulation v3, the results of simulations v1 and v2 are shown in Additional file 1: Figs. S8 and S9, respectively.

The two-sided version of the Wilcoxon signed-rank test was used to test for any difference between multiple normalization methods. After it became evident that MIX$_{\text {minimal}}$ performed best, we used a one-sided version of the Wilcoxon signed-rank test to verify if RMSE and rRMSE are significantly decreased by MIX$_{\text {minimal}}$ compared to all other normalization methods. The resulting *p*-values are listed in Additional file 1: Table S7. Again, MIX$_{\text {minimal}}$ significantly outperformed all other tested methods in RMSE and rRMSE except for PQN in any of the simulations.

We, therefore, conclude that normalizing the sweat volume by the MIX$_{\text {minimal}}$ method reduces the error for the estimated $V$ compared to other tested methods. Compared to PKM, MIX$_{\text {minimal}}$ has the advantage that its performance does not vary if metabolites’ concentration time series can be described with a modified Bateman function (i.e., simulations v1, v2 v3 have little influence on its performance). Therefore, it is especially advantageous if this property cannot be guaranteed.

#### Computational performance

Analysis of metabolomics data sets is usually a computationally exhaustive process. There are several steps in (pre-)processing that need to be executed, many of them lasting for hours. Therefore, computational time can quickly stack to large numbers. Normalization models are no exception to this general rule. As ${n_{\text {metabolites}}}$ in a pharmacokinetic model increases, the time for optimization of pharmacokinetic models may become limiting. Therefore, we investigated the average time for one time series normalization for different methods and different numbers of metabolites.

**Fig. 8:**
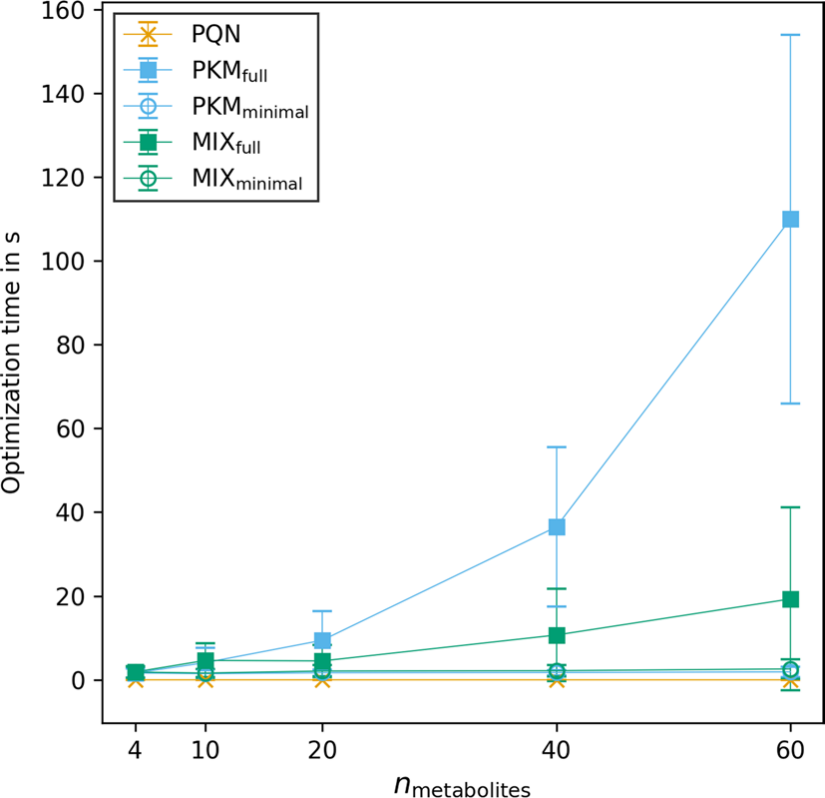
Time in seconds for optimization of one normalization model in simulation v3. The error bars represent the standard deviation of normalization times between 100 replicates

The computational time spent for one optimization step as a function of ${n_{\text {metabolites}}}$ is given in Fig. 8 for simulation v3. It increased for some normalization models, however not for all of them and not equally. Within the investigated range, PQN stays well under 1 second per normalization, whereas with PKM$_{\text {full}}$ the normalization time increases drastically from $1.6 \pm {1.1}\,\hbox {s}$ for a model with 4 metabolites to $110 \pm {44}\,\hbox {s}$ for 60 metabolites. Similar normalization times were observed for MIX$_{\text {full}}$ maxing out at $19 \pm {22}\,\hbox {s}$ for ${n_{\text {metabolites}}}= 60$. In stark contrast to the exponential increase in computational power needed for full models are the minimal models. Their time to optimize stays nearly constant ($< {3}\,\hbox {s}$) within the investigated metabolite range (Additional file 1: Table S8).

Here we demonstrate that MIX$_{\text {minimal}}$ is not only superior to other tested models in terms of its normalization performance but also in terms of computational feasibility. We hypothesize that even data sets with thousands of untargeted metabolites will have a minor impact on its speed.

### Comparison of PQN and MIX

**Fig. 9:**
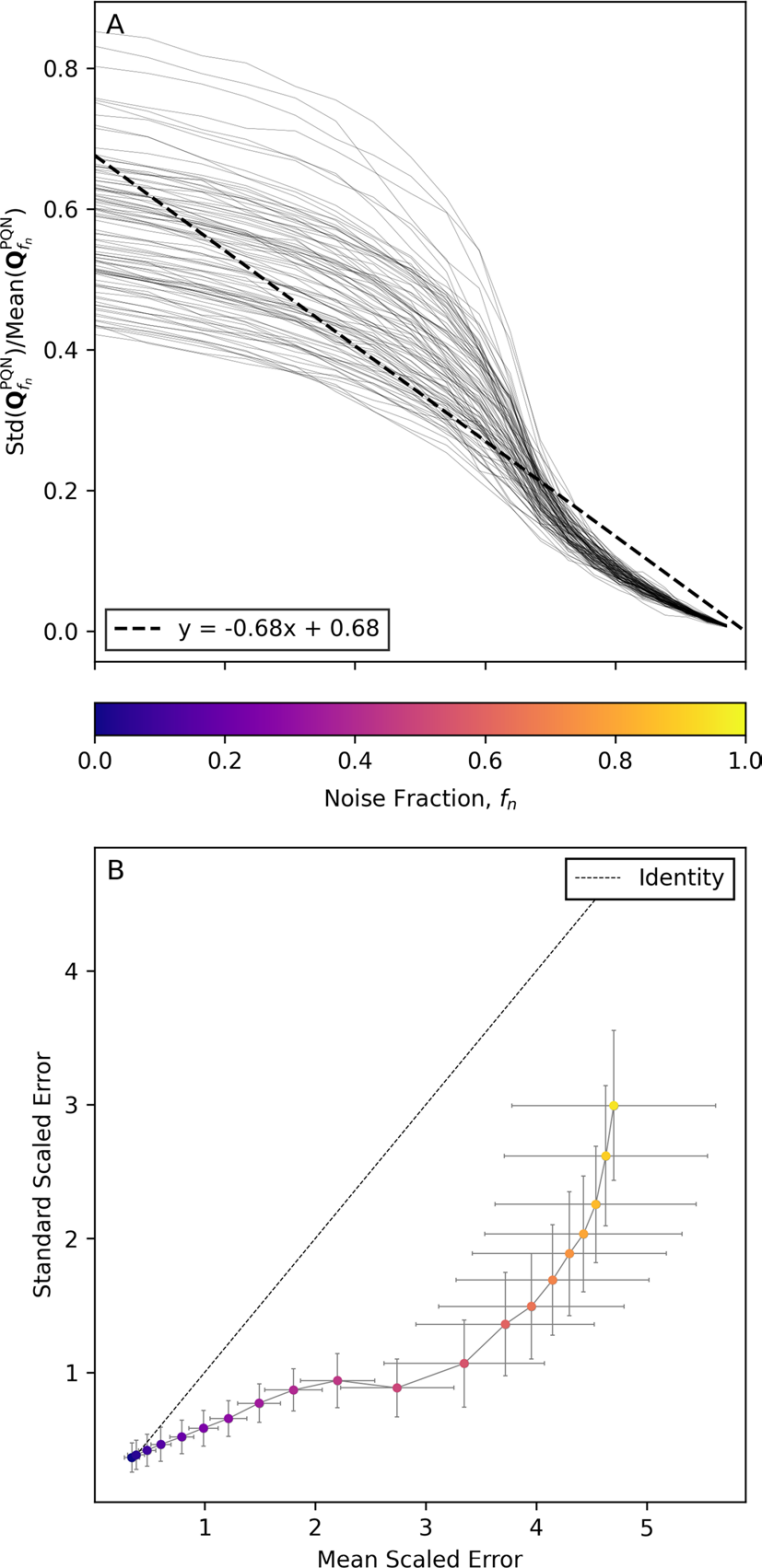
Influence of the fraction of noisy data on the error of PQN calculation. Panel A illustrates the change of the coefficient of variance of $\mathbf {Q}^\text {PQN}$ (Y-axis) as the noise fraction ($f_n$, Y-axis with the same tick labels as the color bar) increases. Panel B shows the error size of calculated $Q^\text {PQN}$ to true $V$ with mean scaling (X-axis) and standard scaling (Y-axis). The color of points relates to the noise fraction as depicted in the color bar

#### Influence of noise on PQN

In untargeted metabolomics, it is often difficult to distinguish between metabolites originating from the actual matrix of interest or from contamination. As PQN includes all untargeted metabolites in its calculation, metabolites stemming from contamination might become a problem as their fold change is independent of the sweat volume, which changes the underlying distributions of quotients. Therefore, we investigated the influence of different fractions of metabolites originating from contamination (i.e., noisy data). Furthermore, we tested if scaling of $\mathbf {Q}^\text {PQN}$ values can counteract errors introduced by noise.

Figure 9A demonstrates the problem of using the probabilistic quotient normalization on noisy raw data. The direction of size effects can still be explained when noise is present, however, absolute values of the size effects decrease. Thus, in Fig. 9A, the coefficient of variation (i.e., the standard deviation over the mean) of ${\textbf {Q}}^\text {PQN}$ is a measure for the average value of the estimated size effect over one synthetically generated time series. As the fraction of noise ($f_n$, X-axis) increases the coefficient of variation decreases drastically and approaches 0 when $f_n \rightarrow 1$.

Figure 9B shows the performance of scaling methods to counteract the reduction of coefficient of variation as described above. The mean scaled error (X-axis) and standard scaled error (Y-axis) as calculated by Eq. 17 are plotted against each other. When $f_n \le 0.05$, mean scaling outperforms standard scaling. However, thereafter the standard scaled $Q^\text {PQN}$ is less erroneous than the mean scaled version.

When incorporating $\mathbf {Q}^\text {PQN}$ values to the MIX model, it is important to correct for errors introduced by noise. As this result shows that standard scaling reduces the detrimental effect of noise on the calculation of $\mathbf {Q}^\text {PQN}$, we used standard scaling throughout the study for MIX normalization. Moreover, this result underlines the good performance of standard scaling in biological data sets [44].

**Fig. 10:**
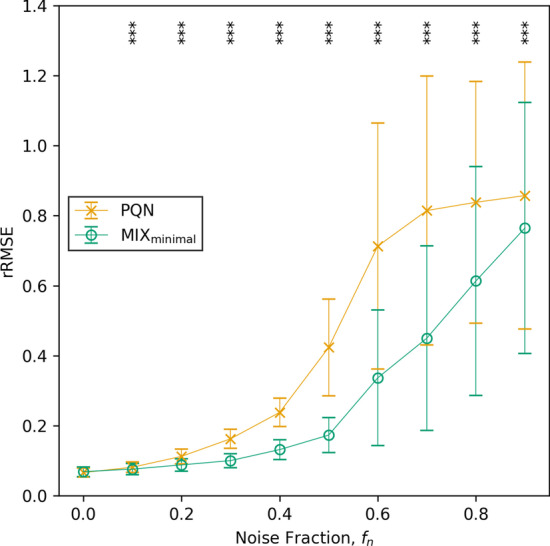
Comparison of the rRMSE of PQN and MIX$_{\text {minimal}}$ on data with different fractions of noise. Significant differences in rRMSE between PQN and MIX$_{\text {minimal}}$ were tested with an one-sided pairwise Wilcoxon signed-rank test

#### Synthetic data simulations with noise

The synthetic data used for the analysis of section "Comparison of PKM and MIX" did not contain any metabolites that are classified as noise, i.e., their $\widetilde{\mathbf {M}}$ is not influenced by size effects (Eq. 15). This, however, is not necessarily a realistic assumption as there are many sources of contaminants in metabolome measurements. Noisy metabolites can be either introduced by biological means (e.g., metabolites that do not originate from sweat but from the surface of the skin in sweat measurements) [45] or by experimental handling [46]. As shown in Fig. 9, this noise in data negatively affects the performance of PQN. Thus, the goodness of PQN in the results of section "Comparison of PKM and MIX" is probably overestimated.

To get a more accurate view of the goodness of normalization of PQN and MIX$_{\text {minimal}}$, we tested their performance on synthetic data with different fractions of noise, $f_n$. In order to do so, we created 100 replicates of synthetic data sampled from real data (i.e., simulation v3) for 10 equidistant noise fractions ranging from $f_n = 0$ to $f_n = 0.9$ with ${n_{\text {metabolites}}}= 60$. In all simulated data, only untargeted metabolites were affected by the introduction of noise, as we assumed that for targeted metabolites (i.e., $\ell = 4$) with known pharmacokinetic behaviour, one can be highly confident that the measurements are not originating from contaminants.

The rRMSE of PQN and MIX$_{\text {minimal}}$ is plotted in Fig. 10. Only when zero noise was present in the synthetic data set MIX$_{\text {minimal}}$ did not improve upon PQN. However, as the noise fraction increased, MIX$_{\text {minimal}}$ significantly outperformed PQN in terms of rRMSE. The *p*-values for all noise fractions are listed in the Additional file 1: Table S9.

The difference of rRMSE between PQN and MIX$_{\text {minimal}}$ in Fig. 10 is related to the difference of mean and standard scaled errors in Fig. 9B. PQN alone cannot utilize the improved performance of standard scaling as $\text {Std}(T(\mathbf {V}))$ has to be known for its calculation (Eq. 17b). However, when normalizing with MIX$_{\text {minimal}}$, $\text {Std}(T(\mathbf {V}))$ can be estimated from the pharmacokinetic part of the model (Eq. 9c) significantly improving its quality.

**Fig. 11:**
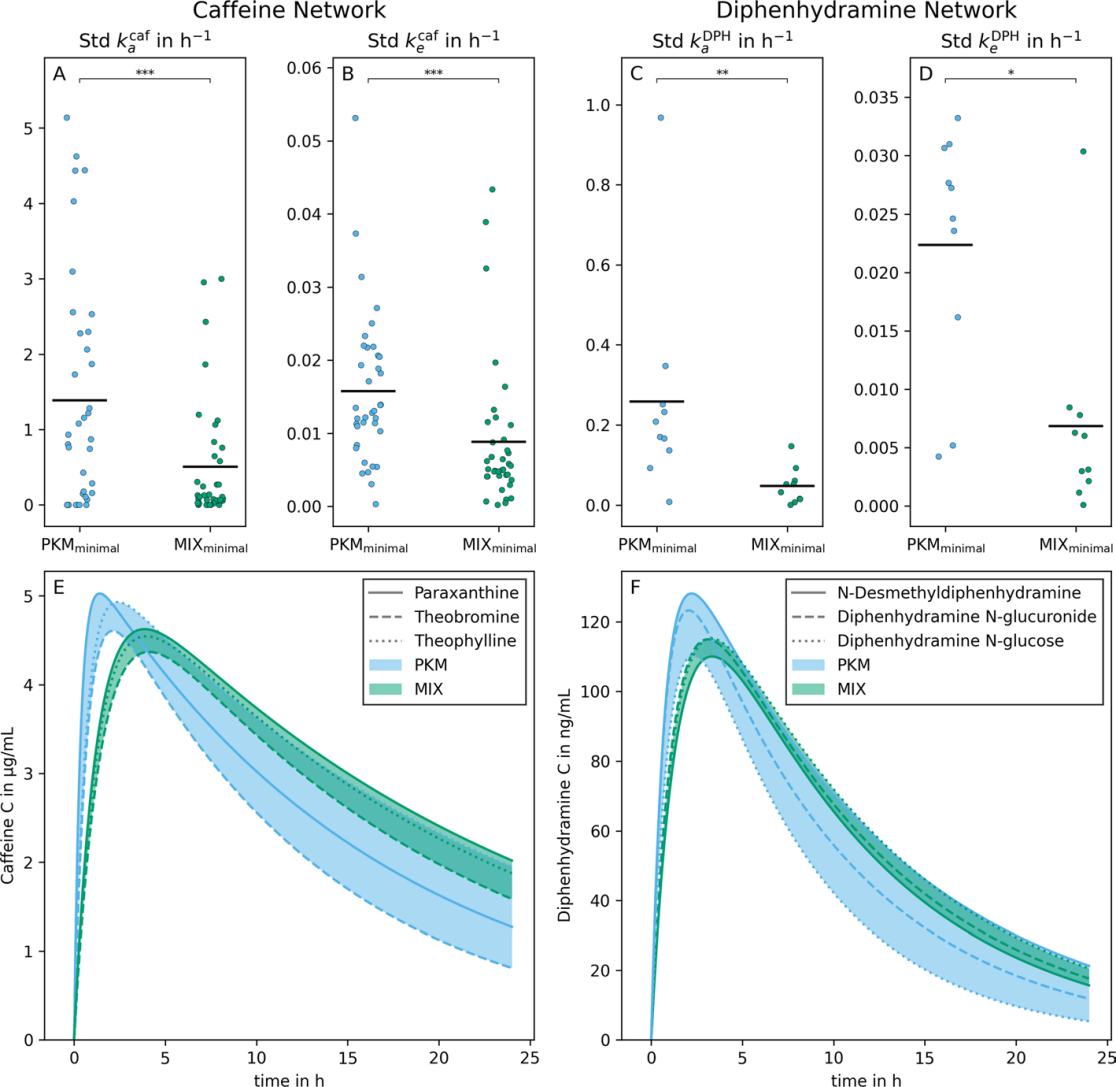
Method validation with finger sweat (left column) and blood plasma (right column) data from Brunmair et al., 2021 [20] and Panitchpakdi et al., 2021 [41] respectively. On panels A to D, the standard deviations of constants of absorption and elimination of caffeine and diphenhydramine ($k_a^\text {caf}$, $k_e^\text {caf}$, $k_a^\text {DPH}$, $k_e^\text {DPH}$) between the three modeled subnetworks are plotted. The number of points per method corresponds to the number of concentrations time series present in both data sets (i.e., 37 and 10 for sweat and plasma, respectively). A one-sided Wilcoxon signed-rank test was used to test for significant differences. Panels E and F show the estimated concentration time series of caffeine and DPH plotted from the three different subnetworks. The lines are named after the second metabolite with a known kinetic present in the subnetwork; however, they all refer to *C* of caffeine and DPH. The colors of curves and the area between them indicate the results from normalization with PKM$_{\text {minimal}}$ or MIX$_{\text {minimal}}$, respectively

### Application to real data

#### Caffeine network

Previously, we identified and quantified four metabolites (caffeine, paraxanthine, theobromine, and theophylline) in a time series after ingesting a single dose of caffeine [20]. To investigate the performance of normalization models on a real finger sweat data set, we split all measured $\widetilde{\mathbf {M}}$ time series into three parts that contained pairs of targeted metabolites each, only one shared by all, namely caffeine (compare Fig. 4 top and bottom network). Subsequently we fitted a PKM$_{\text {minimal}}$ and a MIX$_{\text {minimal}}$ model ($\ell = 2$) with adapted kinetics (Methods section "Real finger sweat metabolome data") through the three sub data sets. Due to the nature of the metabolite subnetworks (Fig. 4 bottom panel) it is possible to calculate two kinetic constants describing the absorption and elimination of caffeine ($k_a^\text {caf}= k_1'$ and $k_e^\text {caf}= k_2' + k_3'$) in all three cases. As the data for all three subnetworks was measured in the same experiment, we can assume that the underlying ground truth of these constants has to be the same. Therefore, by comparing the standard deviation of kinetic constants, it is possible to infer the performance of normalization methods.

In panels A and B of Fig. 11, the standard deviations of fitted kinetic constants within one measured $\widetilde{\mathbf {M}}$ time series are illustrated. Panel A shows that the standard deviations of the absorption constant of caffeine, $k_a^\text {caf}$, of PKM$_{\text {minimal}}$ are significantly larger than of the MIX$_{\text {minimal}}$ model ($p = 5.8\times 10^{-4}, n = 37$, one-sided Wilcoxon signed-rank test). Likewise, a significant decrease in the size of standard deviations of MIX$_{\text {minimal}}$ was found compared to the previously published PKM$_{\text {minimal}}$ model ($p = 1.5~10^{-5}$) for the constant of caffeine elimination, $k_e^\text {caf}$ (panel B, Fig. 11).

In panel E of Fig. 11, one exemplified normalized *C* time series of caffeine in sweat is depicted as fitted for all three subnetworks with PKM$_{\text {minimal}}$ and MIX$_{\text {minimal}}$, respectively. The selected time series illustrates the median of differences in standard deviations between PKM$_{\text {minimal}}$ and MIX$_{\text {minimal}}$ from panels A and B of Fig. 11. The area enclosed by the *C*s of MIX$_{\text {minimal}}$ models is smaller than from PKM$_{\text {minimal}}$.

We emphasize that in our original study, the caffeine degradation directly produces paraxanthine, theobromine, and theophylline; thus, pharmacokinetic parameters $k_2, k_3, k_4$ are explicitly linked [20]. Therefore, the kinetic network resembled specific kinetics of that metabolic pathway (Fig. 4 top panel). In contrast, in previous sections, we assumed that the underlying pathway structure is not known. Thus parameters are not linked, which implies that parameters are less constrained. Yet, in this section, we demonstrated that the fundamental improvement found by switching from PKM to a MIX model can also be translated back again to a more specific metabolic network (Fig. 4 bottom panel). In order to support this argument, we show the applicability of the MIX$_{\text {minimal}}$ normalization method on a real finger sweat data set. The results with real data emphasize the validity of the simulations done on synthetic data sets. They show that, especially when known metabolic networks are small, the MIX$_{\text {minimal}}$ model significantly improves the robustness of normalization and thus kinetic constants inferred from finger sweat time series measurements.

#### Diphenhydramine network

In the original study [41], the authors measured time series abundances in the blood plasma after the application of a single dose of diphenhydramine (DPH). $\widetilde{\mathbf {M}}$ from targeted DPH (known pharmacological constants, known kinetics) as well as untargeted metabolization products (N-desmethyl-DPH, DPH N-glucuronide, DPH N-glucose, known kinetics) and several other untargeted metabolites (unknown kinetics) were reported. Similar to sweat, although less pronounced, plasma also suffers from size effects (i.e., a systematic error in the measurements) introduced by biological means or preanalytical sample handling [47, 48]. Thus, we used the reported data as a second real data set for validation of the performance of MIX$_{\text {minimal}}$. The validation was performed in analogy to the caffeine study where a full network (Additional file 1: Fig. S4) is split into three subnetworks (Additional file 1: Fig. S5, for details see Methods section "Real blood plasma metabolome data").

In panels C and D of Fig. 11, the standard deviations of fitted kinetic constants within one measured $\widetilde{\mathbf {M}}$ and three fitted subnetworks are illustrated. Again, the standard deviations of $k_a^\text {DPH}$ of PKM$_{\text {minimal}}$ are significantly larger than of MIX$_{\text {minimal}}$ ($p = 2,0\times 10^{-3}, n=10$, one-sided Wilcoxon signed-rank test, panel C). A similar significant decrease of the standard deviations are also found for $k_e^\text {DPH}$ ($p = 3.2~10^{-2}$, panel D).

In panel F of Fig. 11, one exemplified normalized *C* time series of DPH in plasma is depicted as fitted for all three subnetworks with PKM$_{\text {minimal}}$ and MIX$_{\text {minimal}}$, respectively. The time series was selected as it is closest to the median of the differences in standard deviations between PKM$_{\text {minimal}}$ and MIX$_{\text {minimal}}$. It is visible that the area enclosed by the *C* resulting from the MIX$_{\text {minimal}}$ model is smaller than from PKM$_{\text {minimal}}$.

This validation illustrates the performance of the normalization models presented in this study on a data set that was measured independently from the development of said methods. The results of the plasma validation study are similar to the results observed for the finger sweat study; again, MIX$_{\text {minimal}}$ improves the robustness (i.e. reduces standard deviations) of size effect normalization.

Even though there is a significant decrease in the standard deviation of $k_e^\text {DPH}$ with MIX$_{\text {minimal}}$ compared to PKM$_{\text {minimal}}$, MIX$_{\text {minimal}}$ also produced an outlier (Fig. 11D). The reason for this outlier is that on rare occasions, MIX$_{\text {minimal}}$ is not able to detect any size effects due to convergence issues (Additional file 1: Fig. S10A). To investigate these results, we performed synthetic data simulations (Additional file 1: Fig. S10B). There, we found that this behaviour of MIX$_{\text {minimal}}$ can be observed when two different $\mathbf {V}$ vectors are applied to $\ell$ and $\ell +$ metabolites. Therefore, we hypothesize that the clearly visible malfunction of MIX$_{\text {minimal}}$ to detect size effects (i.e. the variance of estimated $\mathbf {V}$ is close to 0) gives an indication to scientists that size effects might not be a major concern in such a data set. In this specific blood plasma time series measurement, for example, the size effects might have been too small compared to other error sources to be identified by MIX$_{\text {minimal}}$.

To summarize, with this validation, we show that the generalized normalization models, as implemented in this study, can directly be used for the normalization of real data as long as the modified Bateman function is able to describe the measured kinetics reasonably well and size effects are large enough to be detectable.

## Discussion

In this study, we present a generalized framework for the PKM normalization model, first introduced in reference [20]. Moreover, we extend the existing model to incorporate untargeted metabolite information, dubbed as MIX model. Both models are implemented in Python and are available at GitHub https://github.com/Gotsmy/sweat_normalization.

The quality of normalization methods was tested on synthetic data sets. Synthetic data sets are necessary as it is impossible to obtain validation data without fundamentally changing the (finger) sweat sampling method as described above [20]. However, three different synthetic data generation methods (v1, v2, v3) were employed to ensure that synthetic data sets are as close to real data as possible. We found that when ${n_{\text {metabolites}}}\ge 60$, MIX$_{\text {minimal}}$ performs equally well or better than all other tested normalization methods.

Despite true $V$ values remaining unknown, the real finger sweat data can be used as validation for relative robustness of normalization methods. There, MIX$_{\text {minimal}}$ significantly outperforms PKM$_{\text {minimal}}$. The decreased variance of kinetic constants estimated by MIX$_{\text {minimal}}$ likely originates from the fact that $Q^\text {PQN}$ does not differ much for three subsets as long as sufficiently many ${n_{\text {metabolites}}}= 60$ are present in each subset. On the other hand, as only few data points are used for PKM$_{\text {minimal}}$ optimization, small errors in one of the two targeted metabolites' measured mass have a high potential to change the normalization result.

Additionally, the performance of PKM$_{\text {minimal}}$ and MIX$_{\text {minimal}}$ were compared on a blood plasma data set taken from a study independent of any measurements used for the development of the normalization models. There, we were able to demonstrate the same improvement from PKM$_{\text {minimal}}$ to MIX$_{\text {minimal}}$ in normalization robustness. Moreover, we show that the generalized normalization models as implemented as Python class in this study can be easily used for size effect normalization with little additional coding necessary.

To recapitulate, the proposed MIX$_{\text {minimal}}$ model has several crucial advantages over other tested methods.

- MIX$_{\text {minimal}}$ significantly outperforms PKM$_{\text {minimal}}$ in relative (rRMSE, $-43 \pm {12}\,\%$) and absolute (RMSE, $-73 \pm {10}\,\%$) errors with as little as 60 untargeted metabolites used as additional information (Fig. 7).
- MIX$_{\text {minimal}}$ is invariant to whether untargeted metabolites follow an easily describable kinetic concentration curve (Fig. 6).
- Without noise, MIX$_{\text {minimal}}$ performs equally well as PQN for relative abundances, but additionally, it estimates absolute values of $V$, similar to pharmacokinetic (PKM) models (Fig. 6).
- When noise is present MIX$_{\text {minimal}}$ also outperforms PQN for relative abundances (Fig. 10).
- MIX$_{\text {minimal}}$ performs well in this proof of principle study; moreover, it may be used as a basis for further improvements. Firstly, different, more sophisticated statistical normalization methods (e.g., EigenMS [27]) could be used as input for the PQN part of the model. Secondly, Bayesian priors describing uncertainties of different metabolites could be implemented over the $\lambda$ parameter in a similar fashion as discussed in reference [49].
- Strikingly, the results showed that for all normalization methods tested, the RMSE and rRMSE values flattened once 60 metabolites were present in the original information. This suggested that the presented normalization models, especially MIX$_{\text {minimal}}$, can be applied even for biomatrices or analytical methods with as few as 60 compounds measured.
- Although MIX$_{\text {minimal}}$ was developed especially with sweat volume normalization in mind, it can be easily adapted for other biomatrices, e.g., plasma (Fig. 11).

## Conclusion

In this study, we described and defined the MIX metabolomics time series normalization model and compared it to PKM. Subsequently, we elaborated several advantages of the MIX$_{\text {minimal}}$ model over PKM and previously published normalization methods. We are confident that this will further improve the reliability of metabolomic studies done on finger sweat and other conventional and non-conventional biofluids. However, we acknowledge that a more thorough investigation with data sets of several more quantified metabolites and determined sweat volumes needs to be carried out to assess the full potential of the proposed method.

## Supplementary Information

**Additional file 1**. Supplementary Figures, Tables, and Equations.

## Data Availability

All analysis (except stated otherwise) was performed in Python 3.7 heavily relying on NumPy [50], Pandas [51], and SciPy [32]. Code for simulations, scripts for creation of figures and original and generated data is available on GitHub https://github.com/Gotsmy/sweat_normalization under the GNU GPL version 3 license.

## Abbreviations

$a_\text {sample}$: Sampling skin area
*b*: Part of modified Bateman function
$C, \mathbf {C}$: Underlying concentration (vector)
$$c_0$$: Kinetic parameter
*d*: Kinetic parameter
*F*: Modified Bateman function
$$f_n$$: Noise fraction
*i*: Time point index
*j*: Metabolite index
*k*: Kinetic parameter
$\ell$: Metabolites used for kinetic fitting
$\ell +$: Metabolites not used for kinetic fitting
$$\mathcal {L}$$: Loss
*L*: Loss function
*lag*: Kinetic parameter
$\widetilde{M}, \widetilde{\mathbf {M}}$: Measured mass (vector)
$M^\text {ref}$: Reference mass for PQN
*m*/*z*: Mass over charge ratio
${n_{\text {metabolites}}}$: Number of metabolites
$n_{\text {time points}}$: Number of time points
*p*: *p*-value
$$q_\text {sweat}$$: Sweat rate
$Q^\mathbf {C}$: Median concentration fold change of two samples
$Q^\mathbf {M}$: Median mass fold change of two samples
$Q^\text {PQN}$,$\mathbf {Q}^\text {PQN}$: Normalization quotient (vector) calculated by PQN
R$^2$: Coefficient of determination
rRMSE: Relative measure of goodness of normalization
RMSE: Absolute measure of goodness of normalization
Std: Standard deviation
*T*: Transformation function
*t*: Time
$V$, $\mathbf {V}$: Collected (sweat) volume (vector)
$V^{\text {ref}}$: Reference volume for PQN
Var: Variance
v1, v2, v3: Synthetic data sets
*Z*: Scaling function
$\varvec{\upepsilon }$: Experimental error vector
$\varvec{\uptheta }$: Kinetic parameter vector for fitting
$\lambda$: Loss weighting parameter
$\tau$: Time to collect one sample

## References

[1] Jang M, Costa C, Bunch J, Gibson B, Ismail M, Palitsin V, Webb R, Hudson M, Bailey M (2020). On the relevance of cocaine detection in a fingerprint. Sci Rep.

[2] Delgado-Povedano M, Calderón-Santiago M, de Castro ML, Priego-Capote F (2018). Metabolomics analysis of human sweat collected after moderate exercise. Talanta.

[3] Brunmair J, Bileck A, Stimpfl T, Raible F, Del Favero G, Meier-Menches SM, Gerner C. Metabo-tip: a metabolomics platform for lifestyle monitoring supporting the development of novel strategies in predictive, preventive and personalised medicine. EPMA J. 2021:1–13 10.1007/s13167-021-00241-6 PMC8192631 34188726

[4] Czerwinska J, Jang M, Costa C, Parkin MC, George C, Kicman AT, Bailey MJ, Dargan PI, Abbate V (2020). Detection of mephedrone and its metabolites in fingerprints from a controlled human administration study by liquid chromatography-tandem mass spectrometry and paper spray-mass spectrometry. Analyst.

[5] Calderón-Santiago M, Priego-Capote F, Turck N, Robin X, Jurado-Gámez B, Sanchez JC, De Castro MDL (2015). Human sweat metabolomics for lung cancer screening. Anal Bioanal Chem.

[6] Cui X, Zhang L, Su G, Kijlstra A, Yang P (2021). Specific sweat metabolite profile in ocular Behcet’s disease. Int Immunopharmacol.

[7] Harshman SW, Browder AB, Davidson CN, Pitsch RL, Strayer KE, Schaeublin NM, Phelps MS, O’Connor ML, Mackowski NS, Barrett KN (2021). The impact of nutritional supplementation on sweat metabolomic content: a proof-of-concept study. Front Chem.

[8] Hussain JN, Mantri N, Cohen MM (2017). Working up a good sweat-the challenges of standardising sweat collection for metabolomics analysis. Clin Biochemist Rev.

[9] Harshman SW, Strayer KE, Davidson CN, Pitsch RL, Narayanan L, Scott AM, Schaeublin NM, Wiens TL, Phelps MS, O’Connor ML (2021). Rate normalization for sweat metabolomics biomarker discovery. Talanta.

[10] Kuwayama K, Tsujikawa K, Miyaguchi H, Kanamori T, Iwata YT, Inoue H (2013). Time-course measurements of caffeine and its metabolites extracted from fingertips after coffee intake: a preliminary study for the detection of drugs from fingerprints. Anal Bioanal Chem.

[11] Kuwayama K, Yamamuro T, Tsujikawa K, Miyaguchi H, Kanamori T, Iwata YT, Inoue H (2014). Time-course measurements of drugs and metabolites transferred from fingertips after drug administration: usefulness of fingerprints for drug testing. Forensic Toxicol.

[12] Baker LB (2019). Physiology of sweat gland function: the roles of sweating and sweat composition in human health. Temperature.

[13] Nyein HYY, Bariya M, Tran B, Ahn CH, Brown BJ, Ji W, Davis N, Javey A (2021). A wearable patch for continuous analysis of thermoregulatory sweat at rest. Nat Commun.

[14] Taylor NA, Machado-Moreira CA (2013). Regional variations in transepidermal water loss, eccrine sweat gland density, sweat secretion rates and electrolyte composition in resting and exercising humans. Extreme Physiol Med.

[15] Ando H, Noguchi R. Dependence of palmar sweating response and central nervous system activity on the frequency of whole-body vibration. Scand J Work Environ Health. 2003:216–219. 10.5271/sjweh.724 12828391

[16] Zhong B, Jiang K, Wang L, Shen G. Wearable sweat loss measuring devices: from the role of sweat loss to advanced mechanisms and designs. Adv Sci. 2021:2103257. 10.1002/advs.202103257 PMC8728835 34713981

[17] Harshman SW, Pitsch RL, Smith ZK, O’Connor ML, Geier BA, Qualley AV, Schaeublin NM, Fischer MV, Eckerle JJ, Strang AJ (2018). The proteomic and metabolomic characterization of exercise-induced sweat for human performance monitoring: a pilot investigation. PLoS ONE.

[18] Sonner Z, Wilder E, Heikenfeld J, Kasting G, Beyette F, Swaile D, Sherman F, Joyce J, Hagen J, Kelley-Loughnane N (2015). The microfluidics of the eccrine sweat gland, including biomarker partitioning, transport, and biosensing implications. Biomicrofluidics.

[19] Du Q, Zhang Y, Wang J, Chang J, Wang A, Ren X, Liu B (2022). Quantitative analysis of 17 hypoglycemic drugs in fingerprints using ultra-high-performance liquid chromatography/tandem hybrid triple quadrupole linear ion trap mass spectrometry. Rapid Commun Mass Spectrom.

[20] Brunmair J, Gotsmy M, Niederstaetter L, Neuditschko B, Bileck A, Slany A, Feuerstein ML, Langbauer C, Janker L, Zanghellini J (2021). Finger sweat analysis enables short interval metabolic biomonitoring in humans. Nat Commun.

[21] Filzmoser P, Walczak B (2014). What can go wrong at the data normalization step for identification of biomarkers?. J Chromatogr A.

[22] Singh AS, Masuku MB (2014). Sampling techniques and determination of sample size in applied statistics research: an overview. Int J Econ Commerce Manag.

[23] Choi J, Bandodkar AJ, Reeder JT, Ray TR, Turnquist A, Kim SB, Nyberg N, Hourlier-Fargette A, Model JB, Aranyosi AJ (2019). Soft, skin-integrated multifunctional microfluidic systems for accurate colorimetric analysis of sweat biomarkers and temperature. ACS Sensors.

[24] Kim SB, Koo J, Yoon J, Hourlier-Fargette A, Lee B, Chen S, Jo S, Choi J, Oh YS, Lee G (2020). Soft, skin-interfaced microfluidic systems with integrated enzymatic assays for measuring the concentration of ammonia and ethanol in sweat. Lab Chip.

[25] Ragan TJ, Bailey AP, Gould AP, Driscoll PC (2013). Volume determination with two standards allows absolute quantification and improved chemometric analysis of metabolites by nmr from submicroliter samples. Anal Chem.

[26] Warrack BM, Hnatyshyn S, Ott K-H, Reily MD, Sanders M, Zhang H, Drexler DM (2009). Normalization strategies for metabonomic analysis of urine samples. J Chromatogr B.

[27] Karpievitch YV, Nikolic SB, Wilson R, Sharman JE, Edwards LM (2014). Metabolomics data normalization with eigenms. PLoS ONE.

[28] Dieterle F, Ross A, Schlotterbeck G, Senn H (2006). Probabilistic quotient normalization as robust method to account for dilution of complex biological mixtures. Application in 1h nmr metabonomics. Anal Chem.

[29] Li B, Tang J, Yang Q, Cui X, Li S, Chen S, Cao Q, Xue W, Chen N, Zhu F (2016). Performance evaluation and online realization of data-driven normalization methods used in lc/ms based untargeted metabolomics analysis. Sci Rep.

[30] Di Guida R, Engel J, Allwood JW, Weber RJ, Jones MR, Sommer U, Viant MR, Dunn WB (2016). Non-targeted uhplc-ms metabolomic data processing methods: a comparative investigation of normalisation, missing value imputation, transformation and scaling. Metabolomics.

[31] Macedo AN, Mathiaparanam S, Brick L, Keenan K, Gonska T, Pedder L, Hill S, Britz-McKibbin P (2017). The sweat metabolome of screen-positive cystic fibrosis infants: Revealing mechanisms beyond impaired chloride transport. ACS Cent Sci.

[32] Virtanen P, Gommers R, Oliphant TE, Haberland M, Reddy T, Cournapeau D, Burovski E, Peterson P, Weckesser W, Bright J (2020). Scipy 1.0: fundamental algorithms for scientific computing in python. Nat Methods.

[33] Garrett ER (1994). The bateman function revisited: a critical reevaluation of the quantitative expressions to characterize concentrations in the one compartment body model as a function of time with first-order invasion and first-order elimination. J Pharmacokinet Biopharm.

[34] Brunius C, Shi L, Landberg R (2016). Large-scale untargeted lc-ms metabolomics data correction using between-batch feature alignment and cluster-based within-batch signal intensity drift correction. Metabolomics.

[35] Kvasnička A, Friedecký D, Tichá A, Hyšpler R, Janečková H, Brumarová R, Najdekr L, Zadák Z (2021). SLIDE—Novel Approach to Apocrine Sweat Sampling for Lipid Profiling in Healthy Individuals. International Journal of Molecular Sciences.

[36] Brunmair J, Gotsmy M, Niederstaetter L, Neuditschko B, Bileck A, Slany A, Feuerstein ML, Langbauer C, Janker L, Zanghellini J, et al. Finger sweat analysis enables short interval metabolic biomonitoring in humans. 10.5281/zenodo.5222967. 10.1038/s41467-021-26245-4 PMC8514494 34645808

[37] Chambers MC, Maclean B, Burke R, Amodei D, Ruderman DL, Neumann S, Gatto L, Fischer B, Pratt B, Egertson J (2012). A cross-platform toolkit for mass spectrometry and proteomics. Nat Biotechnol.

[38] Tsugawa H, Ikeda K, Takahashi M, Satoh A, Mori Y, Uchino H, Okahashi N, Yamada Y, Tada I, Bonini P (2020). A lipidome atlas in ms-dial 4. Nat Biotechnol.

[39] Csajka C, Haller C, Benowitz N, Verotta D (2005). Mechanistic pharmacokinetic modelling of ephedrine, norephedrine and caffeine in healthy subjects. Br J Clin Pharmacol.

[40] Kamimori GH, Karyekar CS, Otterstetter R, Cox DS, Balkin TJ, Belenky GL, Eddington ND (2002). The rate of absorption and relative bioavailability of caffeine administered in chewing gum versus capsules to normal healthy volunteers. Int J Pharm.

[41] Panitchpakdi M, Weldon KC, Jarmusch AK, Gentry EC, Choi A, Sepulveda Y, Aguirre S, Sun K, Momper JD, Dorrestein PC, et al. Non-invasive skin sampling detects systemically administered drugs in humans. PloS one, 17(7), e0271794. 10.1371/journal.pone.0271794 PMC9321436 35881585

[42] Panitchpakdi M, Weldon KC, Jarmusch AK, Gentry EC, Choi A, Sepulveda Y, Aguirre S, Sun K, Momper JD, Dorrestein PC, et al. Non-Invasive Skin Sampling Detects Systemically Administered Drugs in Humans. https://gnps.ucsd.edu/ProteoSAFe/status.jsp?task=deee382b163f4441afea5fda4b2a2bcf. Accessed 20 Dec 2021. 10.1371/journal.pone.0271794 PMC9321436 35881585

[43] Wilcoxon F (1945). Individual comparisons by ranking methods. Biometrics Bull..

[44] van den Berg RA, Hoefsloot HC, Westerhuis JA, Smilde AK, van der Werf MJ (2006). Centering, scaling, and transformations: improving the biological information content of metabolomics data. BMC Genomics.

[45] Baker LB, Wolfe AS (2020). Physiological mechanisms determining eccrine sweat composition. Eur J Appl Physiol.

[46] da Silva RR, Vargas F, Ernst M, Nguyen NH, Bolleddu S, Del Rosario KK, Tsunoda SM, Dorrestein PC, Jarmusch AK (2018). Computational removal of undesired mass spectral features possessing repeat units via a kendrick mass filter. J Am Soc Mass Spectrom.

[47] Kamlage B, Maldonado SG, Bethan B, Peter E, Schmitz O, Liebenberg V, Schatz P (2014). Quality markers addressing preanalytical variations of blood and plasma processing identified by broad and targeted metabolite profiling. Clin Chem.

[48] Pinto J, Domingues MRM, Galhano E, Pita C, do Céu Almeida M, Carreira IM, Gil AM. Human plasma stability during handling and storage: impact on nmr metabolomics. Analyst 2014;139(5):1168–77. 10.1039/c3an02188b 24443722

[49] Sheiner LB, Beal SL (1982). Bayesian individualization of pharmacokinetics: simple implementation and comparison with non-Bayesian methods. J Pharm Sci.

[50] Harris CR, Millman KJ, van der Walt SJ, Gommers R, Virtanen P, Cournapeau D, Wieser E, Taylor J, Berg S, Smith NJ, Kern R, Picus M, Hoyer S, van Kerkwijk MH, Brett M, Haldane A, del Río JF, Wiebe M, Peterson P, Gérard-Marchant P, Sheppard K, Reddy T, Weckesser W, Abbasi H, Gohlke C, Oliphant TE (2020). Array programming with NumPy. Nature.

[51] Pandas Development Team, T.: Pandas-dev/pandas: Pandas. 10.5281/zenodo.3509134.

